# *microRNA-142* guards against autoimmunity by controlling T_reg_ cell homeostasis and function

**DOI:** 10.1371/journal.pbio.3001552

**Published:** 2022-02-18

**Authors:** Wei-Le Wang, Ching Ouyang, Natalie M. Graham, Yuankun Zhang, Kaniel Cassady, Estefany Y. Reyes, Min Xiong, Alicia M. Davis, Kathie Tang, Defu Zeng, Mark P. Boldin

**Affiliations:** 1 Irell and Manella Graduate School of Biological Sciences, Beckman Research Institute, City of Hope, Duarte, California, United States of America; 2 Department of Systems Biology, Beckman Research Institute, City of Hope, Duarte, California, United States of America; 3 Center for Informatics, Beckman Research Institute, City of Hope, Duarte, California, United States of America; 4 Department of Computational and Quantitative Medicine, Beckman Research Institute, City of Hope, Duarte, California, United States of America; 5 Department of Diabetes Research, Beckman Research Institute, City of Hope, Duarte, California, United States of America; Children’s Hospital of Philadelphia and The University of Pennsylvania School of Medicine, UNITED STATES

## Abstract

Regulatory T (T_reg_) cells are critical in preventing aberrant immune responses. Posttranscriptional control of gene expression by microRNA (miRNA) has recently emerged as an essential genetic element for T_reg_ cell function. Here, we report that mice with T_reg_ cell–specific ablation of *miR-142* (hereafter *Foxp3*^*Cre*^*miR-142*^*fl/fl*^ mice) developed a fatal systemic autoimmune disorder due to a breakdown in peripheral T-cell tolerance. *Foxp3*^*Cre*^*miR-142*^*fl/fl*^ mice displayed a significant decrease in the abundance and suppressive capacity of T_reg_ cells. Expression profiling of *miR-142*–deficient T_reg_ cells revealed an up-regulation of multiple genes in the interferon gamma (IFNγ) signaling network. We identified several of these IFNγ-associated genes as direct miR-142-3p targets and observed excessive IFNγ production and signaling in *miR-142*–deficient T_reg_ cells. *Ifng* ablation rescued the T_reg_ cell homeostatic defect and alleviated development of autoimmunity in *Foxp3*^*Cre*^*miR-142*^*fl/fl*^ mice. Thus, our findings implicate *miR-142* as an indispensable regulator of T_reg_ cell homeostasis that exerts its function by attenuating IFNγ responses.

## Abstract 2

This study identifies microRNA-142 as a central regulator of the development, homeostasis and function of regulatory T cells. Mechanistically, the miR-142-3p isoform controls regulatory T cell homeostasis and immune tolerance by attenuating IFNγ responses.

## Introduction

Regulatory T (T_reg_) cells are vital in maintaining immune self-tolerance and restraining aberrant immune responses against infections [1,2]. *Foxp3*, an X chromosome–linked member of the forkhead box/winged helix family of transcription factors, is a master regulator of the genetic program that governs development and suppressive activity of T_reg_ cells. Humans and mice that carry loss-of-function *Foxp3* mutations develop a fatal autoimmune disease due to impaired T_reg_ cell activity [3–7]. The majority of T_reg_ cells are generated in the thymus (tT_reg_ cells) through a selection process that favors cells with a strong functional T cell receptor (TCR) avidity toward self-antigens. In contrast, peripheral T_reg_ (pT_reg_) cells arise from naive CD4^+^ T cells upon encounter of non–self-antigens in the context of appropriate cytokine stimulation [8–10]. Harnessing the power of T_reg_ cells to control immunological responses has a great potential for human therapy because, on one hand, T_reg_ cells can promote transplantation tolerance, but on the other, can hinder antitumor immunity.

Posttranscriptional regulation of gene expression by microRNA (miRNA), a class of small (approximately 22 nucleotides) noncoding RNA, recently emerged as a critical genetic element that is essential for T_reg_ cell function. T_reg_ cell–specific knockouts (KOs) of either *Drosha* or *Dicer* genes, encoding 2 endonucleases required for mature miRNA generation from precursor transcripts, phenocopy mice with *Foxp3* ablation, and develop severe systemic autoimmunity because of a defect in T_reg_ cell activity [11–13]. Furthermore, deletion of *Dicer* at the double positive (DP) thymocyte stage in mice significantly diminished the frequency of tT_reg_ cells, suggesting that miRNA-dependent gene control is also required for normal development of T_reg_ cells [11]. Thus, the current pressing challenge in the field is to determine how specific miRNA gene(s) exert control of the T_reg_ cell genetic program. The present report addresses this goal by examining the role of *miR-142* in T_reg_ cell development and function.

*miR-142* is predominantly expressed in cells of hematopoietic origin and encodes 2 abundant mature miRNA molecules—miR-142-5p and miR-142-3p—which arise from the opposite strands of the hairpin-like miR-142 precursor. Using genetic loss-of-function studies, *miR-142* was previously implicated in the regulation of ontogenesis and function of several immune cell types. Our earlier report determined that deletion of this miRNA gene in mice results in aberrant B lymphopoiesis and impaired humoral immunity [14]. In addition, *miR-142*–deficient mice develop thrombocytopenia stemming from defective megakaryocyte maturation [15] and exhibit dysregulation of dendritic cell (DC) function [16]. Disruption of 2 *miR-142* paralog genes in zebra danio using zinc-finger nucleases was reported to cause aberrant neutrophil differentiation [17]. In the T-cell compartment, *miR-142* is required for the homeostasis of peripheral T effector (T_eff_) cells, but is apparently dispensable for conventional T (T_conv_) cell development in the thymus [14,18,19]. A recent study by Anandagoda and colleagues has demonstrated that *miR-142* is essential for the immunosuppressive activity of T_reg_ cells, but failed to reveal a significant role for this miRNA in T_reg_ cell development and homeostasis [20]. The authors suggest that posttranscriptional repression of the cAMP-hydrolyzing enzyme *Pde3b* by miR-142-5p isoform plays a key role in the regulation of the T_reg_ cell suppressive function.

Here, we show that mice with a conditional deletion of *miR-142* in T_reg_ cells develop severe autoimmune disease due to a profound defect in T_reg_ cell homeostasis and function. In addition, our findings suggest that *miR-142* plays an important role in tT_reg_ cell development. We have determined that the miR-142-3p isoform and its capacity to silence multiple interferon gamma (IFNγ)-associated genes play a critical role in mediating the regulatory activity of *miR-142* in T_reg_ cells. Global ablation of IFNγ rescues the T_reg_ cell defect and autoimmunity in *Foxp3*^*Cre*^*miR-142*^*fl/fl*^ mice, thus providing further evidence for the essential role of the *miR-142*-IFNγ signaling pathway in the regulation of T_reg_ cell homeostasis and function.

## Results

### *miR-142* is dynamically expressed in T cells, and its global ablation results in a T_reg_ cell defect

Our efforts to define the role of *miR-142* in the regulation of T-cell tolerance stem from an unexpected observation that global *miR-142* ablation results in a marked T_reg_ cell defect. We found that germline *miR-142* KO mice display a significant drop in T_reg_ cell numbers in both thymus and secondary lymphoid organs (S1A–S1C Fig), indicating that *miR-142* plays an important role in T_reg_ cell development and homeostasis. The observed defect was specific to T_reg_ cells, because thymic development of T_conv_ cells was largely normal in the germline *miR-142* KO mice [14,18,19]. Our expression profiling experiments revealed that the mature miR-142-3p isoform is abundantly expressed in thymic and pT_reg_ cells, whereas the mature miR-142-5p isoform is present in significantly lower amounts (S1D Fig). The level of miR-142-3p expression in T_reg_ cells is roughly comparable to the expression of this miRNA in naive and activated T_eff_ cells. Of note, *miR-142* is dynamically expressed during T-cell development: its abundance gradually increases following T-cell maturation in the thymus and reaches a peak at the single positive (SP) thymocyte stage; however, mature T cells that egress from the thymus into periphery display a somewhat reduced *miR-142* expression in comparison to SP thymocytes (S1D Fig).

### Lethal autoimmune disease in mice with T_reg_ cell–specific *miR-142* deletion

**Fig 1:**
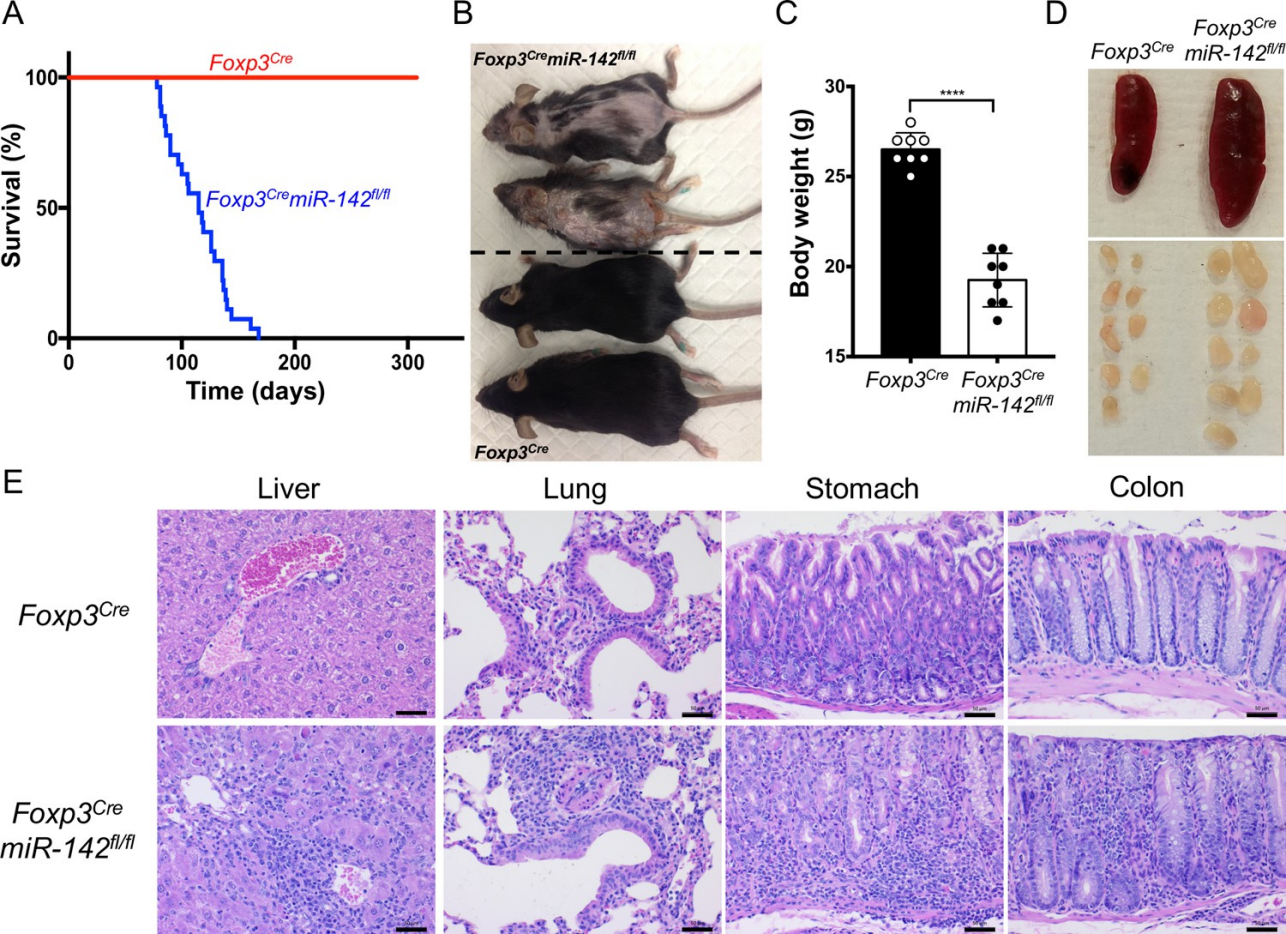
Fatal autoimmune disorder in mice with T_reg_ cell–specific disruption of *miR-142*. (**A**) Kaplan–Meier survival curves of *Foxp3*^*Cre*^ and *Foxp3*^*Cre*^*miR-142*^*fl/fl*^ mice (*n* = 27 per group) (*P* < 0.0001). (**B**) Photograph of 14-week-old female *Foxp3*^*Cre*^ (lower 2) and *Foxp3*^*Cre*^*miR-142*^*fl/fl*^ (upper 2) mice. Note smaller body size and severe dermatitis in *Foxp3*^*Cre*^*miR-142*^*fl/fl*^ mice. (**C**) Body weight comparison of 8- to 10-week-old male *Foxp3*^*Cre*^ and *Foxp3*^*Cre*^*miR-142*^*fl/fl*^ mice (*n* = 8 per group). (**D**) Representative images of spleen and peripheral lymph nodes from *Foxp3*^*Cre*^ (left) and *Foxp3*^*Cre*^*miR-142*^*fl/fl*^ (right) mice. (**E**) HE staining of liver, lung, stomach, and colon tissue sections from *Foxp3*^*Cre*^ and *Foxp3*^*Cre*^*miR-142*^*fl/fl*^ mice. Note massive accumulation of leukocytes in *Foxp3*^*Cre*^*miR-142*^*fl/fl*^ tissues. Scale bar, 50 μm. Results are shown as mean ± SD. *P* values were determined by log-rank test (A) or 2-tailed Student *t* test (C); ****, *P* < 0.0001. The underlying raw data can be found in S1 Data file. HE, hematoxylin–eosin; SD, standard deviation; T_reg_, regulatory T.

To determine the role of *miR-142* in T_reg_ cell function, we created mice with a T_reg_ cell–specific ablation of this miRNA (hereafter *Foxp3*^*Cre*^*miR-142*^*fl/fl*^ mice). *Foxp3*^*Cre*^ deleter/reporter mice [21] that were used to generate the T_reg_ cell–specific excision of *miR-142* conditional allele (*miR-142*^*fl/fl*^) express Cre recombinase as a fusion protein with yellow fluorescent protein (YFP) under the control of endogenous *Foxp3* promoter, providing an effective way to purify T_reg_ cells by flow cytometry. As expected, CD4^+^YFP^+^ T_reg_ cells isolated from *Foxp3*^*Cre*^*miR-142*^*fl/fl*^ mice were virtually devoid of mature miR-142-3p, whereas expression of this miRNA in CD4^+^ T_conv_ cells remained unchanged (S1E Fig). Despite appearing normal at birth, *Foxp3*^*Cre*^*miR-142*^*fl/fl*^ mice exhibited an apparent failure to thrive: they had a very short life span (Fig 1A), were smaller in size (Fig 1B), and had significantly lower body weight than wild-type (WT; *Foxp3*^*Cre*^) littermates (Fig 1C). Gross morphological analysis revealed that *Foxp3*^*Cre*^*miR-142*^*fl/fl*^ mice developed a systemic lymphoproliferative autoimmune disorder that was characterized by significant lymphadenopathy (Fig 1D and S1J Fig), mild splenomegaly (Fig 1D, S1F and S1H Fig), thymic involution (S1G and S1I Fig), dermatitis (Fig 1B), and massive immune cell infiltration into various peripheral organs (Fig 1E). Taken together, the phenotypic findings from *Foxp3*^*Cre*^*miR-142*^*fl/fl*^ mice resemble the autoimmune pathology observed in mice with severe T_reg_ cell defects (e.g., *scurfy* mutants or *Foxp3* KO mice [3–7]), suggesting that *miR-142* is involved in the regulation of T_reg_ cell homeostasis or stability.

### T_reg_ cell defect and aberrant T-cell activation in *Foxp3*^*Cre*^*miR-142*^*fl/fl*^ mice

**Fig 2:**
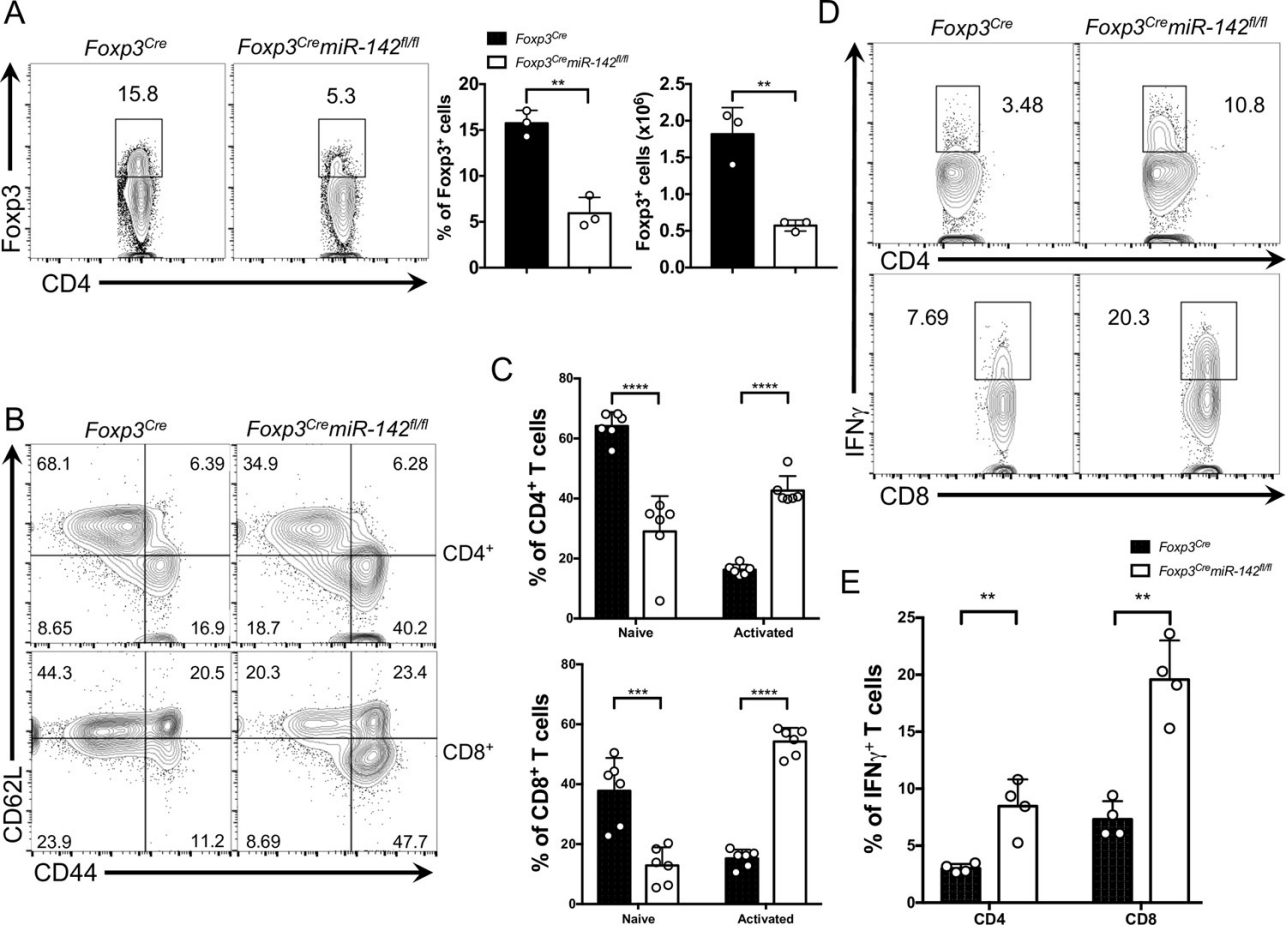
*miR-142* is required for maintenance of T_reg_ cell–mediated immune tolerance. (**A**) Left panel, FACS analysis of splenic lymphocytes from 12-week-old *Foxp3*^*Cre*^ and *Foxp3*^*Cre*^*miR-142*^*fl/fl*^ mice with anti-CD4 and anti-Foxp3 specific antibodies. Numbers indicate percentage of CD4^+^Foxp3^+^ T_reg_ cells in the gate. Right panel, frequency and absolute number of T_reg_ cells in *Foxp3*^*Cre*^ and *Foxp3*^*Cre*^*miR-142*^*fl/fl*^ spleens (*n* = 3 per group). (**B**) FACS analysis of splenic CD4^+^ and CD8^+^ T cells from 8- to 10-week-old *Foxp3*^*Cre*^ and *Foxp3*^*Cre*^*miR-142*^*fl/fl*^ mice with anti-CD44 and anti-CD62L specific antibodies. Numbers indicate percentage of cells in the quadrants. (**C**) Frequency of naive (CD44^−^CD62L^+^) and activated (CD44^+^CD62L^−^) splenic CD4^+^ (top) and CD8^+^ (bottom) T lymphocytes isolated from 8- to 10-week-old *Foxp3*^*Cre*^ and *Foxp3*^*Cre*^*miR-142*^*fl/fl*^ mice (*n* = 6 per group). (**D**) Intracellular FACS analysis of IFNγ production in splenic CD4^+^ (top) and CD8^+^ (bottom) T cells. Numbers indicate percentage of IFNγ^+^ T cells in the gate. (**E)** Frequency of IFNγ expressing splenic CD4^+^ and CD8^+^ T cells isolated from 8- to 10-week-old *Foxp3*^*Cre*^ and *Foxp3*^*Cre*^*miR-142*^*fl/fl*^ mice (*n* = 4 per group). Results are shown as mean ± SD. *P* values were calculated using 2-tailed Student *t* test. **, *P* < 0.01; ***, *P* < 0.001; ****, *P* < 0.0001. The underlying numerical raw data can be found in S1 Data file. The underlying flow cytometry raw data can be found at the Figshare repository. FACS, fluorescence activated cell sorting; IFNγ, interferon gamma; SD, standard deviation; T_reg_, regulatory T.

In agreement with this notion, we observed a marked reduction in T_reg_ cell population in the *Foxp3*^*Cre*^*miR-142*^*fl/fl*^ spleen (Fig 2A). Furthermore, the absolute number of thymic T_reg_ cells in *Foxp3*^*Cre*^*miR-142*^*fl/fl*^ mice was also significantly lower, despite a slight increase in the frequency of T_reg_ cells in the atrophied thymus (S2A and S2B Fig). Because T_reg_ cells play a crucial role in suppressing self-destructive responses elicited by autoreactive T cells, we examined the activation status of peripheral T cells in *Foxp3*^*Cre*^*miR-142*^*fl/fl*^ mice by flow cytometry. In comparison to WT mice, the number of CD4^+^ and CD8^+^ T cells with memory/effector phenotype (CD44^hi^CD62L^lo^) was significantly higher in the spleen and lymph nodes of *Foxp3*^*Cre*^*miR-142*^*fl/fl*^ mice, whereas the pool of naive (CD44^lo^CD62L^hi^) CD4^+^ and CD8^+^ T cells in the KO animals diminished considerably (Fig 2B and 2C, S2C and S2D Fig). *Foxp3*^*Cre*^*miR-142*^*fl/fl*^ mice exhibited an expansion of peripheral CD4^+^ and CD8^+^ T-cell compartments (S2G Fig), most likely due to a failure of *miR-142*–deficient T_reg_ cells to suppress proliferation of hyperactivated T lymphocytes. In addition, both CD4^+^ and CD8^+^ T cells isolated from *Foxp3*^*Cre*^*miR-142*^*fl/fl*^ mice displayed a sharp increase in production of IFNγ (Fig 2D and 2E, S2E and S2F Fig), a pro-inflammatory cytokine that promotes type 1 T helper cell 1 (Th1) responses. In contrast, no obvious changes in interleukin (IL)-4 (secreted by Th2 cells) or IL-17 (secreted by Th17 cells) production by splenic CD4^+^ T cells were observed in *Foxp3*^*Cre*^*miR-142*^*fl/fl*^ animals (S2H Fig). Thus, our findings suggest that conditional *miR-142* deletion impairs T_reg_ cell function and subsequently drives severe dysregulation of T_eff_ responses.

### *miR-142* is essential for the homeostasis and suppressive activity of T_reg_ cells

**Fig 3:**
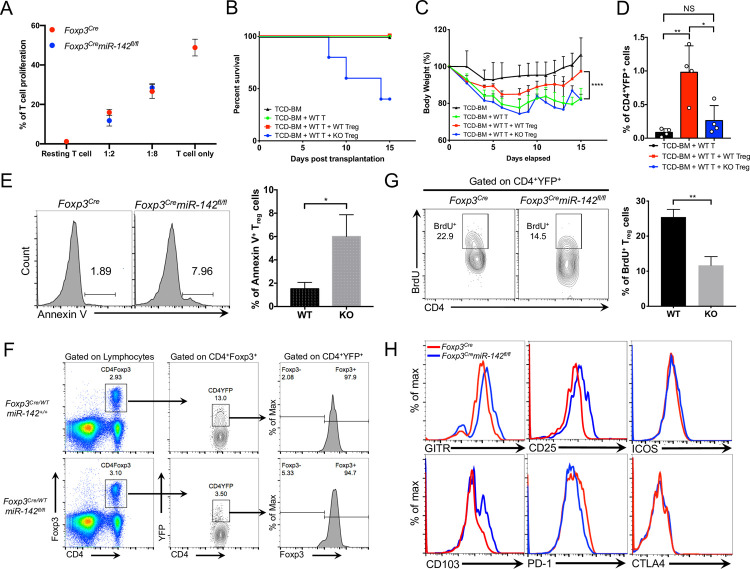
*miR-142* ablation impairs suppressive function and homeostasis of T_reg_ cells. (**A**) *in vitro* T_reg_ suppression assay. Purified CD4^+^ T_conv_ cells were loaded with CTV dye and incubated with FACS-sorted T_reg_ cells from *Foxp3*^*Cre*^ (red dots) and *Foxp3*^*Cre*^*miR-142*^*fl/fl*^ (blue dots) spleens (*n* = 3) in the presence of beads coated with anti-CD3 and anti-CD28 specific antibodies. After 3 days, the rate of T_conv_ cell proliferation was determined by flow cytometry as dilution of the CTV dye. Several T_reg_ to T_conv_ cell ratios were analyzed as indicated in the graph. Unstimulated T_conv_ cells were used as control. (**B**) Survival of mice in aGVHD model. Lethally irradiated BALB/c recipient mice were transplanted with T-cell–depleted bone marrow from C57BL/6J mice (TCD-BM), Thy1.2^+^CD4^+^ T cells from C57BL/6J spleens (WT T), and CD4^+^YFP^+^ T_reg_ cells derived from either *Foxp3^Cre^* (WT) or *Foxp3^Cre^miR-142^fl/fl^* (KO) spleens (*P* = 0.0096; *n* = 5 per group). (**C**) Body weight analysis of mice in aGVHD model. Mouse grouping as in B (*n* = 5 per group). (**D**) Frequency of donor *Foxp3*^*Cre*^ (WT) or *Foxp3*^*Cre*^*miR-142*^*fl/fl*^ (KO) CD4^+^YFP^+^ T_reg_ cells in spleens of BALB/c recipient mice on day 17 after BMT (*n* = 4 for all groups). (**E**) Left panel, FACS analysis of activation-induced cell death in splenic *Foxp3*^*Cre*^ and *Foxp3*^*Cre*^*miR-142*^*fl/fl*^ T_reg_ cells. Apoptotic T_reg_ cells were detected by Annexin V staining upon stimulation with anti-CD3 (1 μg/ml) specific antibodies for 48 hours. Numbers indicate percentage of Annexin V^+^ cells. Right panel, the frequency of Annexin V^+^ *Foxp3*^*Cre*^ (WT) and *Foxp3*^*Cre*^*miR-142*^*fl/fl*^ (KO) T_reg_ cells (*n* = 3 per group). (**F**) FACS analysis of CD4^+^Foxp3^+^ T_reg_ cells (left panels), YFP-Cre expression in CD4^+^Foxp3^+^ T_reg_ cells (middle panels), and Foxp3 expression in CD4^+^YFP^+^ *miR-142*–sufficient and *miR-142*–deficient T_reg_ cells (right panels) from female *Foxp3*^*Cre/WT*^*miR-142*^*+/+*^ and *Foxp3*^*Cre/WT*^*miR-142*^*fl/fl*^ mice. (**G**) Left panel, intracellular FACS analysis of BrdU incorporation into T_reg_ cells isolated from *Foxp3*^*Cre*^ and *Foxp3*^*Cre*^*miR-142*^*fl/fl*^ spleens. FACS plots were pregated on CD4^+^YFP^+^ T_reg_ cells, and numbers indicate percentage of cells in the gate. Right panel, the frequency of BrdU^+^ T_reg_ cells in *Foxp3*^*Cre*^ (WT) and *Foxp3*^*Cre*^*miR-142*^*fl/fl*^ (KO) spleens 16 hours after BrdU injection (1mg i.p.) (*n* = 3 per group). (**H**) FACS analysis of cell surface markers on T_reg_ cells isolated from *Foxp3*^*Cre*^ (red) and *Foxp3*^*Cre*^*miR-142*^*fl/fl*^ (blue) spleens with anti-GITR, anti-CD25, anti-CD103, anti-PD-1, anti-CTLA-4, and anti-ICOS specific antibodies. Histograms were pregated on CD4^+^YFP^+^ T_reg_ cells. Results are shown as mean ± SD. *P* values were calculated using log-rank test (B), 1-way ANOVA (C), and 2-tailed Student *t* test (D, E, G). *, *P* < 0.05; **, *P* < 0.01; ****, *P* < 0.0001; NS, not significant. The underlying numerical raw data can be found in S1 Data file. The underlying flow cytometry raw data can be found at the Figshare repository. aGVHD, acute graft-versus-host disease; BMT, bone marrow transplantation; CTV, cell trace violet; FACS, fluorescence activated cell sorting; KO, knockout; SD, standard deviation; T_reg_, regulatory T; YFP, yellow fluorescent protein; WT, wild-type.

To determine how *miR-142* ablation affects suppressive function of T_reg_ cells, we cocultured purified *miR-142*–deficient and *miR-142*–sufficient T_reg_ cells together with WT T_conv_ cells that were induced to proliferate by antigen receptor stimulation. We found that *miR-142*–deficient T_reg_ cells exhibited a comparable capacity to restrain the proliferation of activated T_conv_ cells as *miR-142*–sufficient T_reg_ cells in this classical *in vitro* T_reg_ cell suppression assay (Fig 3A, S3A Fig). Similar *in vitro* findings were previously reported for *miR-146a* and *miR-181a/b-1*, two other miRNAs that are implicated in the regulation of T_reg_ cell activity [22,23]. Nevertheless, adoptive transfer of *miR-142*–deficient T_reg_ cells failed to attenuate development of systemic inflammatory syndrome in a mouse model of acute graft-versus-host disease (aGVHD). Lethally irradiated *BALB/c* mice (H2^d^) transplanted with allogeneic CD4^+^ T_conv_ cells from *C57BL/6* donors (H2^b^) rapidly developed sublethal aGVHD that was manifested by hunched posture, severe diarrhea, and significant weight loss in the host mice (Fig 3B and 3C). The concomitant transfer of WT T_reg_ cells together with allogeneic donor T cells reduced the severity of aGVHD symptoms, as indicated by diminished diarrhea and less pronounced body weight loss in the host mice (Fig 3C). In contrast, adoptive transfer of *miR-142*–deficient T_reg_ cells failed to protect the host mice from the development of aGVHD and resulted in mortality, perhaps by exacerbating the inflammatory response (Fig 3B and 3C). The failure of *miR-142*–deficient T_reg_ cells to suppress aGVHD indicated an essential role of *miR-142* in the regulation of T_reg_ cell suppressive activity *in vivo*. In addition, a significantly lower frequency of donor *miR-142*–deficient T_reg_ cells in the spleens of host mice 17 days posttransplantation (Fig 3D) suggested the survival or homeostatic defect of *miR-142*–deficient T_reg_ cells.

To test whether *miR-142* ablation affects survival of T_reg_ cells, we compared induction of apoptosis in *miR-142*–sufficient and *miR-142*–deficient T_reg_ cells in response to TCR stimulation. We found that purified splenic *miR-142* KO T_reg_ cells were more prone to activation-induced cell death (Fig 3E), although resting T_reg_ cells from *Foxp3*^*Cre*^*miR-142*^*fl/fl*^ spleen displayed no obvious difference in Annexin V staining (S3B Fig). To further establish the cell-intrinsic role of *miR-142* in T_reg_ cell homeostasis, we enumerated *miR-142*–deficient T_reg_ cells in *Foxp3*^*Cre/WT*^*miR-142*^*fl/fl*^ heterozygous female mice. Due to random X chromosome inactivation of *Foxp3*^*Cre*^ allele, female *Foxp3*^*Cre/WT*^*miR-142*^*fl/fl*^ mice generate both YFP^−^ *miR-142*–sufficient and YFP^+^ *miR-142*–deficient T_reg_ cells. Our analysis revealed a marked reduction in the frequency of splenic YFP^+^ *miR-142*–deficient T_reg_ cells in the mosaic mice (Fig 3F, S3C Fig), indicating that *miR-142* plays a cell-autonomous role in T_reg_ cell homeostasis. In addition, *in vivo* labeling experiments using 5-bromo-2-deoxyuridine (BrdU) revealed that *miR-142*–deficient T_reg_ cells have almost 2-fold lower proliferation capacity than WT T_reg_ cells (Fig 3G). Collectively, these findings implicate *miR-142* in the regulation of homeostatic maintenance and proliferation of T_reg_ cells.

Analysis of cell surface markers on pT_reg_ cells from *Foxp3*^*Cre*^*miR-142*^*fl/fl*^ mice by flow cytometry suggested that *miR-142*–deficient T_reg_ cells display an activated/effector phenotype [24]. Consistent with this notion, *miR-142*–deficient T_reg_ cells exhibited an up-regulation of several T-cell activation markers, including glucocorticoid-induced tumor necrosis factor receptor family-related gene (GITR), interleukin-2 receptor α (CD25), and integrin α_E_ (CD103) (Fig 3H, S3D Fig). On the other hand, the levels of programmed death-1 (PD-1) receptor, which protects T_reg_ cells from apoptosis [25], were significantly lower on *miR-142*–deficient T_reg_ cells (Fig 3H, S3D Fig), although expression of other immune checkpoint molecules such as cytotoxic T-lymphocyte associated protein 4 (CTLA4) and inducible T-cell co-stimulator (ICOS) was not affected.

### Specific deletion of *miR-142* in T_reg_ cells results in global derepression of miR-142-3p targets

**Fig 4:**
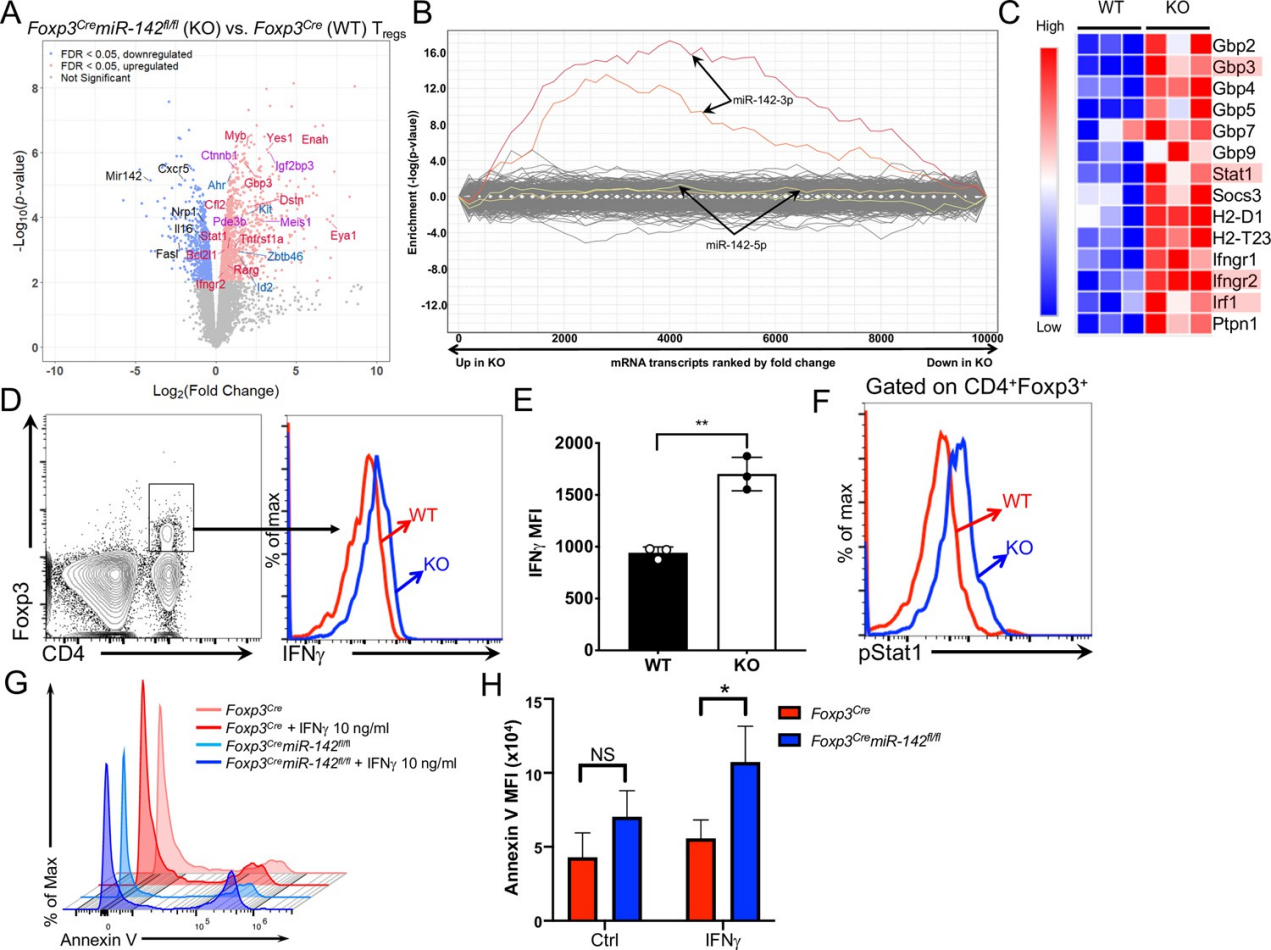
Global derepression of miR-142-3p targets and dysregulated IFNγ signaling in *miR-142*–deficient T_reg_ cells. (**A**) Volcano plot showing statistical significance versus magnitude of change for visualizing the distribution of differentially expressed genes. Selected *miR-142* targets (red for -3p, blue for -5p, and purple for both) and other genes of functional interest (black; including *miR-142*) are labeled. (**B**) Global derepression of miR-142-3p target genes in *miR-142*–deficient T_reg_ cells. SylArray analysis of 3′ UTRs of differentially expressed transcripts in *miR-142*–deficient T_reg_ cells for miRNA SCSs. Enrichment *P* values for each miRNA SCS are plotted on the y-axis against the ranked gene list on the x-axis (the most up-regulated genes in *miR-142*–deficient T_reg_ cells are toward the left, while the most down-regulated genes are toward the right). Plots corresponding to two 7-mer seeds of miR-142-3p are highlighted in red and orange, and two 7-mer seeds of miR-142-5p are depicted in green and yellow. (**C**) Heat map of expression profiles of IFNγ-associated genes in *Foxp3*^*Cre*^ (WT) and *Foxp3*^*Cre*^*miR-142*^*fl/fl*^ (KO) T_reg_ cells (*n* = 3 per group). Genes highlighted in red are putative targets of miR-142-3p. (**D–F**) Intracellular FACS analysis of IFNγ production (**D**) and Stat1(Y701) phosphorylation (**F**) in *Foxp3*^*Cre*^ (red line) and *Foxp3*^*Cre*^*miR-142*^*fl/fl*^ (blue line) CD4^+^Foxp3^+^ T_reg_ cells. (**E**) MFI of IFNγ expression in *Foxp3*^*Cre*^ (WT) and *Foxp3*^*Cre*^*miR-142*^*fl/fl*^ (KO) T_reg_ cells (*n* = 3 per group). (**G**) FACS analysis of Annexin V-stained splenic CD4^+^YFP^+^ T_reg_ cells from *Foxp3*^*Cre*^ (WT) or *Foxp3*^*Cre*^*miR-142*^*fl/fl*^ (KO) mice. Splenocytes were incubated for 24 hours in cell culture in the presence or absence of IFNγ (10 ng/ml) prior to flow cytometry analysis. (**H**) MFI of Annexin V staining on T_reg_ cells from *Foxp3*^*Cre*^ (WT) *or Foxp3*^*Cre*^*miR-142*^*fl/fl*^ (KO) spleens with or without IFNγ treatment (10 ng/ml). Results are shown as mean ± SD. *P* values were calculated using 2-tailed Student t test. *, *P* < 0.05; **, *P* < 0.01; NS, not significant. The underlying numerical raw data can be found in S1 Table and S1 Data file. The underlying flow cytometry raw data can be found at the Figshare repository. FACS, fluorescence activated cell sorting; IFNγ, interferon gamma; KO, knockout; MFI, mean fluorescence intensity; SCS, seed complementary sequence; SD, standard deviation; T_reg_, regulatory T; WT, wild-type.

To uncover the molecular mechanism by which *miR-142* controls T_reg_ cell function, we performed global transcriptome analysis of splenic CD4^+^YFP^+^ *miR-142*–sufficient and *miR-142*–deficient T_reg_ cells using RNA sequencing (RNA-seq). We identified a total of 1,520 genes showing statistically significant change in expression due to *miR-142* ablation, 988 of which were up-regulated and 532 of which were down-regulated in *miR-142*–deficient T_reg_ cells (Fig 4A, S1 Table). Analysis of differentially expressed genes in *miR-142*–deficient T_reg_ cells with the SylArray and ToppFun software algorithms [26,27] revealed a significant enrichment of miR-142-3p (85 out of 429 annotated target genes), but not miR-142-5p, direct targets among the up-regulated genes (Fig 4B), suggesting that impaired homeostasis and function of T_reg_ cells in *Foxp3*^*Cre*^*miR-142*^*fl/fl*^ mice are most likely driven by the loss of mature miR-142-3p expression. This conclusion aligns well with significantly more abundant expression of miR-142-3p in T_reg_ cells (S1D Fig) and a large number of previously published observations from a broad spectrum of *miR-142*–deficient immune cell types [14–18,28–30]. Nevertheless, the possibility that miR-142-5p contributes to the regulation of T_reg_ cell biology (as was recently proposed by Anandagoda and colleagues [20]) either by controlling the expression of a limited number of target mRNAs or by exerting its regulatory effect entirely at the translational level cannot be excluded, although our results suggest that the T_reg_ cell defect in *Foxp3*^*Cre*^*miR-142*^*fl/fl*^ mice is primarily mediated by miR-142-3p.

Pathway enrichment analysis of the differentially expressed genes identified significant overrepresentation of biological processes that are associated with innate and adaptive immune responses (S2 Table). In line with these findings, *miR-142*–deficient T_reg_ cells display an up-regulated expression of diverse cytokine, chemokine, and immune receptor genes (S4A Fig). One potential caveat of this finding is that the aberrant inflammation observed in *Foxp3*^*Cre*^*miR-142*^*fl/fl*^ mice can conceivably induce pro-inflammatory changes in the gene expression of T_reg_ cells. In addition, expression of several T_reg_ cell signature genes was altered by the loss of *miR-142* (S4B Fig). Interestingly, the transcripts up-regulated in *miR-142*–deficient T_reg_ cells were enriched for genes from the IFNγ signaling network according to the Enrichr software algorithm [31] and the Gene Set Enrichment Analysis (GSEA) [32] (Fig 4C, S4C Fig, S2 Table). In agreement with our bioinformatic findings, we found that *miR-142*–deficient T_reg_ cells produced significantly more IFNγ in comparison with WT cells (Fig 4D and 4E, S4D Fig) and displayed dysregulation of IFNγ signaling, manifested by a substantial increase in Stat1 activation (Fig 4F, S4E Fig). Because *miR-142* deletion impairs T_reg_ cell homeostasis, we examined the effect of IFNγ stimulation on T_reg_ cell survival and found that IFNγ treatment significantly enhanced apoptosis of *miR-142*–deficient T_reg_ cells *in vitro* (Fig 4G and 4H).

### *Ifng* ablation rescues the T_reg_ homeostatic defect and alleviates development of the systemic autoimmune disorder in *Foxp3*^*Cre*^*miR-142*^*fl/fl*^ mice

**Fig 5:**
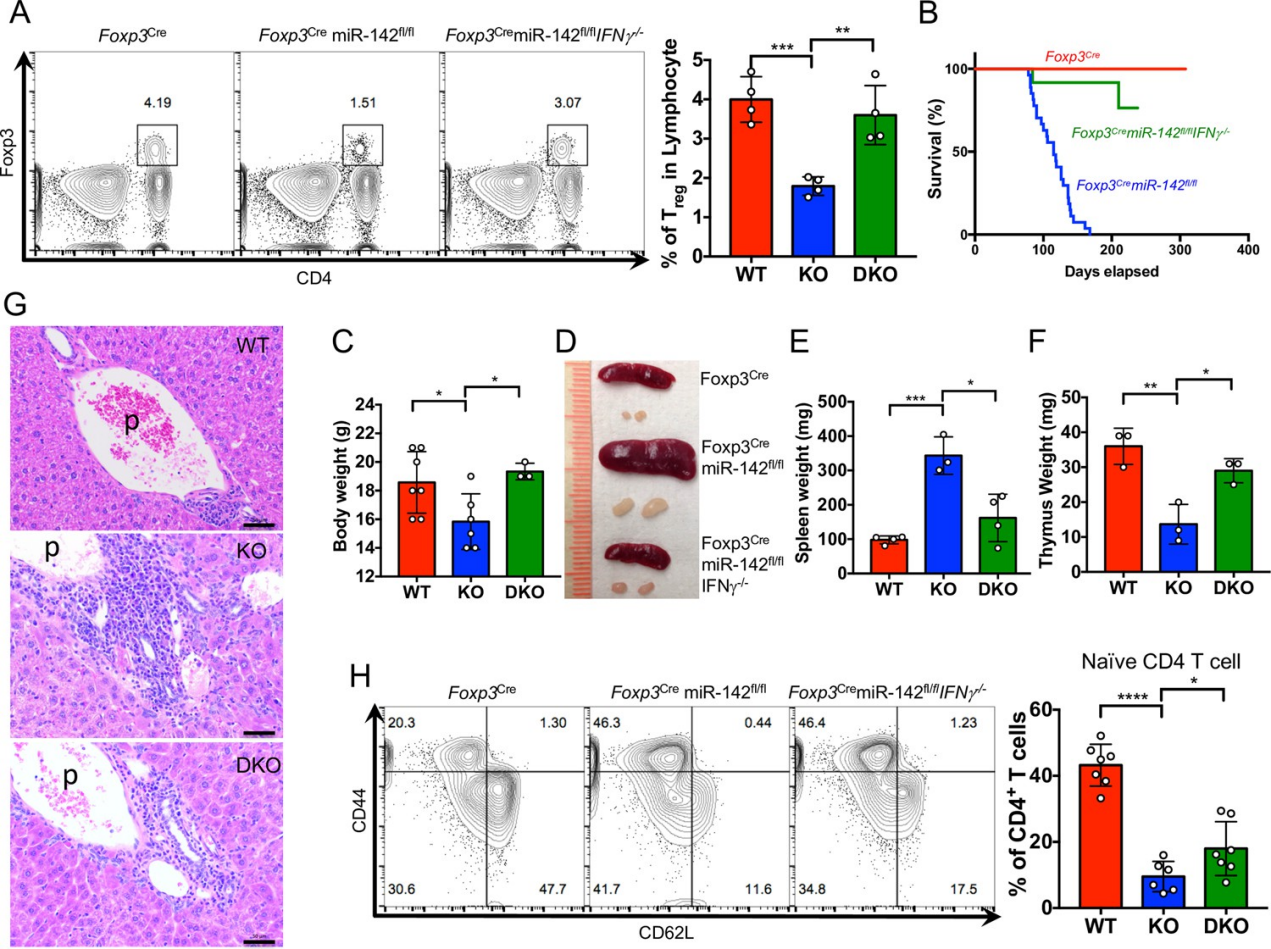
Blockade of IFN*γ* production rescues the T_reg_ cell homeostatic defect and alleviates systemic autoimmunity in *Foxp3*^*Cre*^*miR-142*^*fl/fl*^ mice. (**A**) Left panel, FACS analysis of splenic T_reg_ cells from 12- to 15-week-old *Foxp3*^*Cre*^ (WT), *Foxp3*^*Cre*^*miR-142*^*fl/fl*^ (KO), and *Foxp3*^*Cre*^*miR-142*^*fl/fl*^*Ifng*^−/−^ (DKO) mice (*n* = 4 per group). Foxp3^+^CD4^+^ T_reg_ cells are gated and numbers indicate the percentage of cells in the gate. Right panel, frequency of CD4^+^Foxp3^+^ T_reg_ cells in *Foxp3*^*Cre*^ (WT), *Foxp3*^*Cre*^*miR-142*^*fl/fl*^ (KO), and *Foxp3*^*Cre*^*miR-142*^*fl/fl*^*Ifng*^−/−^ (DKO) spleens. (**B**) Kaplan–Meier survival curves for *Foxp3*^*Cre*^ (red line; *n* = 27), *Foxp3*^*Cre*^*miR-142*^*fl/fl*^ (blue line; *n* = 27), and *Foxp3*^*Cre*^*miR-142*^*fl/fl*^*Ifng*^−/−^ (green line; *n* = 12) mice. (**C**) Body weight comparison of 7- to 9-week-old female *Foxp3*^*Cre*^ (WT, red bar, *n* = 7), *Foxp3*^*Cre*^*miR-142*^*fl/fl*^ (KO, blue bar, *n* = 6), and *Foxp3*^*Cre*^*miR-142*^*fl/fl*^*Ifng*^−/−^ (DKO, green bar, *n* = 3) mice. (**D**) Representative images of spleen and inguinal lymph nodes from 14-week-old *Foxp3*^*Cre*^, *Foxp3*^*Cre*^*miR-142*^*fl/fl*^, and *Foxp3*^*Cre*^*miR-142*^*fl/fl*^*Ifng*^−/−^ mice. (**E**) Spleen weights in 12- to 15-week-old *Foxp3*^*Cre*^ (WT, red bar, *n* = 4), *Foxp3*^*Cre*^*miR-142*^*fl/fl*^ (KO, blue bar, *n* = 3), and *Foxp3*^*Cre*^*miR-142*^*fl/fl*^*Ifng*^−/−^ (DKO, green bar, *n* = 4) mice. (**F**) Thymus weights (1 lobe) in 6- to 8-week-old *Foxp3*^*Cre*^ (WT, red bar), *Foxp3*^*Cre*^*miR-142*^*fl/fl*^ (KO, blue bar), and *Foxp3*^*Cre*^*miR-142*^*fl/fl*^*Ifng*^−/−^ (DKO, green bar) mice (*n* = 3 per group). (**G**) HE staining of liver tissue sections from *Foxp3*^*Cre*^ (WT), *Foxp3*^*Cre*^*miR-142*^*fl/fl*^ (KO), and *Foxp3*^*Cre*^*miR-142*^*fl/fl*^*Ifng*^−/−^ (DKO) mice. Note diminished infiltration of leukocytes into liver tissue around portal vein area in DKO mice. Scale bar, 50 μm; p, portal vein. (**H**) Left panel, FACS analysis of CD44 and CD62L expression in splenic CD4^+^ T cells from 7- to 15-week-old *Foxp3*^*Cre*^, *Foxp3*^*Cre*^*miR-142*^*fl/fl*^, and *Foxp3*^*Cre*^*miR-142*^*fl/fl*^*Ifng*^−/−^ mice. Numbers indicate percentage of cells in the quadrants. Right panel, frequency of CD44^−^CD62L^+^ naive CD4^+^ T cell in *Foxp3*^*Cre*^ (WT, *n* = 7), *Foxp3*^*Cre*^*miR-142*^*fl/fl*^ (KO, *n* = 6), and *Foxp3*^*Cre*^*miR-142*^*fl/fl*^*Ifng*^−/−^ (DKO, *n* = 7) spleens. Results are shown as mean ± SD. *P* values were calculated using 2-tailed Student *t* test. *, *P* < 0.05; **, *P* < 0.01; ***, *P* < 0.001; ****, *P* < 0.0001. WT, *Foxp3*^*Cre*^; KO, *Foxp3*^*Cre*^*miR-142*^*fl/fl*^; DKO, *Foxp3*^*Cre*^*miR-142*^*fl/fl*^*Ifng*^−/−^. The underlying numerical raw data can be found in S1 Data file. The underlying flow cytometry raw data can be found at the Figshare repository. DKO, double knockout; FACS, fluorescence activated cell sorting; HE, hematoxylin–eosin; IFNγ, interferon gamma; KO, knockout; SD, standard deviation; WT, wild-type.

To determine how dysregulated IFN**γ** signaling impacts the homeostasis of *miR-142*–deficient T_reg_ cells, we generated *Foxp3*^*Cre*^*miR-142*^*fl/fl*^*Ifng*^−/−^ double KO mice by crossing *Foxp3*^*Cre*^*miR-142*^*fl/fl*^ mice with *Ifng*^−/−^ mice. Intriguingly, our analysis revealed a complete rescue of the T_reg_ homeostatic defect in the double KO mice (Fig 5A). Furthermore, *Ifng* deletion alleviated development of the systemic lymphoproliferative and fatal autoimmune disease in *Foxp3*^*Cre*^*miR-142*^*fl/fl*^ mice. This conclusion was supported by observations of improved survival (Fig 5B), body weight normalization (Fig 5C) and marked rescue of the lymphadenopathy (Fig 5D), splenomegaly (Fig 5D and 5E), and thymic involution (Fig 5F) defects in *Foxp3*^*Cre*^*miR-142*^*fl/fl*^*Ifng*^−/−^ mice. Of note, the double KO mice displayed an abatement of tissue inflammation, evidenced by a significant decrease in the amount of immune cell infiltration into peripheral tissues (Fig 5G). These morphological changes correlated with a modest increase in the number of naive T_conv_ cells in the periphery of *Foxp3*^*Cre*^*miR-142*^*fl/fl*^*Ifng*^−/−^ mice (Fig 5H), suggesting that the *miR-142*-IFN**γ** signaling axis is primarily important for maintaining T_reg_ cell homeostasis. Despite the restoration of T_reg_ cell frequency in *Foxp3*^*Cre*^*miR-142*^*fl/fl*^*Ifng*^−/−^ mice, the remaining hyperactivation of CD4^+^ T_conv_ cells in the double KO mice implies a potential role for *miR-142* in the regulation of T_reg_ cell immunosuppressive activity.

### miR-142-3p targets multiple IFNγ-associated genes

**Fig 6:**
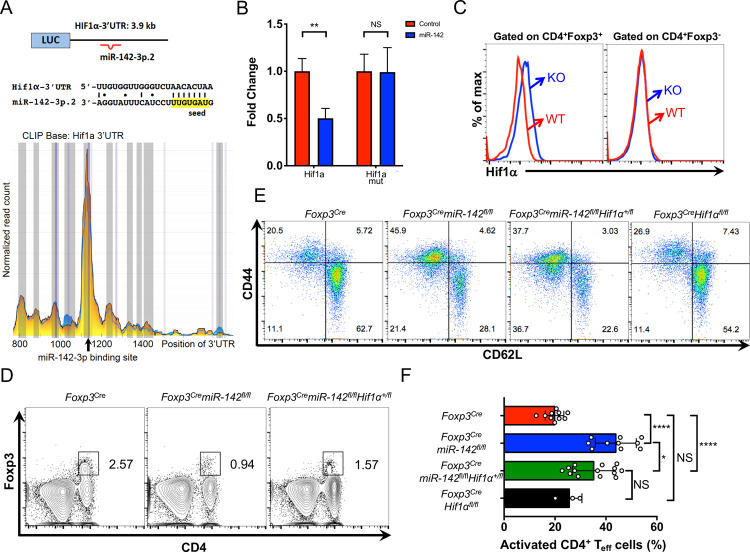
Lowering the *Hif1a* gene dose partially restores the normal size of T_reg_ population and attenuates hyperactivation of peripheral T cells in *Foxp3*^*Cre*^*miR-142*^*fl/fl*^ mice. (**A**) Diagram (top panel) and sequence alignment (middle panel) of miR-142-3p putative binding site in the *Hif1a* 3′ UTR. HITS-CLIP analysis (bottom panel) of Ago2 binding sites in the *Hif1a* 3′ UTR in activated CD4^+^ T cells (blue plot, WT; yellow plot, *miR-155* KO). Note the peak of Ago2 binding activity that corresponds to the predicted miR-142-3p binding site (depicted by arrow). (**B**) Validation of *Hif1a* as direct miR-142-3p target by the 3′ UTR luciferase reporter assay. Relative expression of WT and miR-142-3p seed mutated *Hif1a* 3′ UTR reporter constructs upon cotransfection with either miR-142 precursor expressing plasmid or empty vector control. Expression of WT *Hif1a* 3′ UTR reporter in the presence of empty vector was arbitrarily set to 1. (**C**) Intracellular FACS analysis of Hif1α expression in CD4^+^Foxp3^+^ and CD4^+^Foxp3^-^ T cells from *Foxp3*^*Cre*^ (red line) and *Foxp3*^*Cre*^*miR-142*^*fl/fl*^ (blue line) spleens. (**D**) FACS analysis of splenic lymphocytes from 12- to 18-week-old *Foxp3*^*Cre*^, *Foxp3*^*Cre*^*miR-142*^*fl/fl*^, and *Foxp3*^*Cre*^*miR-142*^*fl/fl*^*Hif1a*^*+/fl*^ mice with anti-CD4 and anti-Foxp3 specific antibodies. Foxp3^+^CD4^+^ T_reg_ cells are gated, and numbers indicate the percentage of cells in the gate. (**E**) FACS analysis of CD44 and CD62L expression in splenic CD4^+^ T cells from *Foxp3*^*Cre*^, *Foxp3*^*Cre*^*miR-142*^*fl/fl*^, *Foxp3*^*Cre*^*miR-142*^*fl/fl*^*Hif1a*^*+/fl*^, and *Foxp3*^*Cre*^*Hif1a*^*fl/fl*^ mice. Numbers indicate percentage of cells in the quadrants. (**F**) Frequency of activated (CD44^+^CD62L^−^) splenic CD4^+^ T cells in *Foxp3*^*Cre*^ (*n* = 10), *Foxp3*^*Cre*^*miR-142*^*fl/fl*^ (*n* = 10), *Foxp3*^Cre^miR-142^fl/fl^*Hif1a*^+/fl^ (*n* = 14), and *Foxp3*^Cre^*Hif1a*^fl/fl^ (*n* = 3) mice. Results are shown as mean ± SD. *P* values were calculated using 2-tailed Student *t* test. *, *P* < 0.05;**, *P* < 0.01; ****, *P* < 0.0001; NS, not significant. WT, *Foxp3*^*Cre*^; KO, *Foxp3*^*Cre*^*miR-142*^*fl/fl*^. The underlying numerical raw data can be found in S1 Data file. The underlying flow cytometry raw data can be found at the Figshare repository. FACS, fluorescence activated cell sorting; *Hif1a*, hypoxia-induced factor 1 alpha; KO, knockout; SD, standard deviation; WT, wild-type.

Several of the IFNγ-associated genes that were derepressed in *miR-142*–deficient T_reg_ cells, including *Ifngr2*, *Stat1*, *Irf1*, and *Gbp3*, are predicted by the TargetScan algorithm [33] as putative direct targets of miR-142-3p (Fig 4C, S5A Fig). We validated these bioinformatic predictions by examining the high-throughput sequencing of RNAs isolated by cross-linking immunoprecipitation (HITS-CLIP) database that was previously compiled by Rudensky and colleagues [34]. In activated CD4^+^ T cells, 2 of the 4 (*Ifngr2* and *Gbp3)* target genes showed detectible peaks of Ago2 binding activity in their respective 3′ UTRs, corresponding to predicted binding sites for miR-142-3p (S5A Fig). An extended analysis of the HITS-CLIP database revealed hypoxia-induced factor 1 alpha (*Hif1a*) as a potential miR-142-3p target (Fig 6A). Our interest in this transcription factor was prompted by a previous report that implicated *Hif1a* in the regulation of T_reg_ cell homeostasis and suppressive function through the control of IFNγ expression [35]. Using 3′ UTR luciferase reporter assay, we confirmed the capacity of miR-142-3p to attenuate *Hif1a and Ifngr2* expression by binding directly to evolutionary conserved seed sequences in their respective 3′ UTRs (Fig 6B, S5B Fig). In line with these findings, we detected a significant increase in Hif1α and IFNγR2 protein levels in *miR-142*–deficient T_reg_ cells, whereas Hif1α expression in CD4^+^ T_conv_ cells remained unchanged (Fig 6C, S5C and S5D Fig). Of note, we did not observe changes in *Hif1a* mRNA expression in T_reg_ cells lacking *miR-142* expression.

In addition, we examined the capacity of miR-142-3p to regulate the expression of phosphodiesterase 3b (*Pde3b*), which was recently identified as a critical miR-142-5p target gene modulating T_reg_ cell activity [20]. In agreement with the previously published results, we detected an approximately 3-fold increase of *Pde3b* mRNA expression in *miR-142*–deficient T_reg_ cells (S1 Table). Analysis of the *Pde3b* 3′ UTR by the TargetScan algorithm revealed 2 putative poorly conserved miR-142-5p binding sites and a single predicted poorly conserved miR-142-3p binding site (S5E Fig). Using the 3′ UTR luciferase reporter assay, we determined that overexpression of *miR-142* precursor can modestly attenuate *Pde3b* expression. Interestingly, we observed that mutation of the miR-142-3p binding site in the *Pde3b* 3′ UTR can completely negate the silencing effect of *miR-142* on the *Pde3b* reporter (S5B and S5F Fig). Thus, our findings have uncovered *Pde3b* as a novel miR-142-3p target gene, providing additional evidence for the critical role of miR-142-3p isoform in the regulation of T_reg_ cell biology.

### *Hif1a* haploinsufficiency partially rescues the T_reg_ cell homeostasis defect in *Foxp3*^*Cre*^*miR-142*^*fl/fl*^ mice

Next, we sought to determine the contribution of the *miR-142-Hif1a* axis in T_reg_ cell homeostasis and function. We lowered the *Hif1a* gene dose in *miR-142*–deficient T_reg_ cells by crossing *Foxp3*^*Cre*^*miR-142*^*fl/fl*^ mice with mice carrying a conditional *Hif1a* allele (*Hif1a*^*fl/fl*^). The resultant *Foxp3*^*Cre*^*miR-142*^*fl/fl*^*Hif1a*^*+/fl*^ mice expressed normal levels of Hif1α protein in T_reg_ cells (S6A Fig) and displayed a partial rescue of T_reg_ cell abundance (Fig 6D, S6B Fig). Furthermore, *Foxp3*^*Cre*^*miR-142*^*fl/fl*^*Hif1a*^*+/fl*^ mice exhibited a partial reduction in the frequency of activated/effector CD4^+^ T cells in the periphery (Fig 6E and 6F). Nevertheless, *Hif1a* haploinsufficiency in *Foxp3*^*Cre*^*miR-142*^*fl/fl*^ mice failed to ameliorate the lethal autoimmunity (S6C Fig), nor did *Hif1a* deletion restore the suppressive capacity (S6F and S6G Fig) of *miR-142*-deficient T_reg_ cells and attenuate the peripheral T_eff_ cell hyperactivation (S6H Fig), perhaps because of a failure to effectively rescue aberrant IFN**γ** production and signaling in T_reg_ cells (S6D and S6E Fig).

## Discussion

Although miRNA-mediated posttranscriptional control of gene expression is recognized as crucial for T_reg_ cell development and function [11–13], our understanding of how specific miRNA genes govern T_reg_ cell responses is incomplete. Here, we report that mice with T_reg_ cell–specific *miR-142* deletion develop a fatal systemic autoimmune disease due to a severe defect in T_reg_ cell homeostasis and suppressive activity. Furthermore, we found that constitutive *miR-142* ablation results in aberrant thymic T_reg_ cell development. Thus, our findings have uncovered an indispensable role for *miR-142* in the control of T_reg_ cell–mediated immunological tolerance. We propose that *miR-142* plays a dominant role among miRNAs involved in the regulation of T_reg_ cell activity because the phenotype of *Foxp3*^*Cre*^*miR-142*^*fl/fl*^ mice closely resembles the autoimmune pathology observed in mice with global disruption of miRNA biogenesis in T_reg_ cells [11–13]. This notion should be confirmed by further conditional KO studies of several miRNA genes that were previously implicated in the regulation of T_reg_ cell function, including *miR-146a*, *miR-155*, *and miR-27* [22,36,37].

The role of *miR-142* in T_reg_ cell function was previously examined by Anandagoda and colleagues using a conditional KO mouse model [20]. In contrast with the findings from this report, we determined that deletion of miR-142-3p and not miR-142-5p (as proposed by Anandagoda and colleagues) is the major driver of the T_reg_ cell defect and subsequent systemic autoimmunity in *Foxp3*^*Cre*^*miR-142*^*fl/fl*^ mice. This conclusion is strongly supported by the observed global derepression of miR-142-3p target genes in *miR-142*–deficient T_reg_ cells, whereas the levels of the majority of miR-142-5p targets did not significantly change. Our inference of a critical role for miR-142-3p in T_reg_ cells is well aligned with the fact that miR-142-3p is more abundantly expressed in T_reg_ cells than miR-142-5p and a large body of literature that assigns the main regulatory role in immune cells to miR-142-3p [14–18,28–30]. Our data revealing that miR-142-3p can directly bind and regulate the phosphodiesterase *Pde3b* gene, through which, as Anandagoda and colleagues suggest [20], *miR-142* controls T_reg_ cell immunosuppressive activity, further validates miR-142-3p as the key miR-142 isoform in T_reg_ cells.

Our findings indicate that miR-142-3p controls T_reg_ cell homeostasis and function by attenuating IFNγ production and signaling. Interestingly, dysregulation of IFNγ responses is increasingly recognized as a critical factor that negatively impacts T_reg_ cell activity. For example, excessive IFNγ production was reported to drive functional “fragility” of T_reg_ cells in the context of antitumor immunity [38]. Additionally, conditional deletion of the E3 ubiquitin ligase von Hippel–Lindau (*Vhl*) gene was shown to impair T_reg_ cell function through IFNγ dysregulation [35]. Moreover, unrestrained *Stat1* activation and subsequent IFNγ production by *Socs1*-deficient T_reg_ cells was linked to a severe failure of immunological tolerance [22]. Finally, a loss of functional activity by *Dicer*-deficient T_reg_ cells was associated with excessive IFNγ production [13].

miRNAs, despite eliciting a moderate effect on the expression of their target genes, often have a significant impact on cellular physiology through coordinated and coherent targeting of multiple key molecules in a signaling cascade [39]. In agreement with this notion, we found that *miR-142* is predicted to control expression of several IFNγ-associated genes, including *Ifngr2*, *Gbp3*, *Stat1*, *Irf1*, and *Hif1a* and validated some of these as *bona fide* miR-142-3p targets. We propose that coordinated derepression of these target genes in *miR-142*–deficient T_reg_ cells drives the observed dysregulation of IFNγ responses. In support of this hypothesis, we found that genetic blockade of IFNγ production rescues the homeostatic defect in *miR-142*–deficient T_reg_ cells and prevents development of systemic lymphoproliferative and autoimmune disorder in *Foxp3*^*Cre*^*miR-142*^*fl/fl*^ mice. Despite a complete restoration of normal T_reg_ cell frequency in *Foxp3*^*Cre*^*miR-142*^*fl/fl*^*Ifng*^−/−^ mice, the partial rescue of peripheral T_eff_ cell hyperactivation in these mice suggests a possibility that *miR-142*–mediated control of T_reg_ cell function is not limited to the attenuation of IFNγ signaling and likely involves additional molecular targets. Another caveat of our genetic epistasis experiments using *Foxp3*^*Cre*^*miR-142*^*fl/fl*^*Ifng*^−/−^ mice is a potential non–cell-autonomous effect of IFNγ deletion on *miR-142*–deficient T_reg_ cells. Because we found a significant IFNγ up-regulation in both *miR-142*–deficient T_reg_ cells and *miR-142*–sufficient T_eff_ cells in *Foxp3*^*Cre*^*miR-142*^*fl/fl*^ mice, the global IFNγ ablation might potentially impact *miR-142*–deficient T_reg_ cell function in cell-extrinsic manner via changes in their IFNγ-rich inflammatory environment. Future analysis of *miR-142*–sufficient and *miR-142*–deficient T_reg_ cells from animals that lack IFNγ expression such as *Foxp3*^*Cre*^*miR-142*^*fl/fl*^*Ifng*^−/−^ and female *Foxp3^Cre/+^miR-142*^*fl/fl*^ mice will be required to corroborate the intrinsic effect of IFNγ on *miR-142*-deficient T_reg_ cells and uncover the additional signaling pathways through which *miR-142* mediates its regulatory functions in T_reg_ cells.

Our investigation of *Hif1a*, a validated miR-142-3p target, revealed an important role for the *miR-142-Hif1a* axis in the regulation of T_reg_ cell homeostasis. We observed that lowering the *Hif1a* gene dose in *miR-142*–deficient T_reg_ cells partially rescued the T_reg_ homeostatic defect and modestly reduced the hyperactivation of peripheral T_eff_ cells in *Foxp3*^*Cre*^*miR-142*^*fl/fl*^ mice. The role suggested by our findings for *Hif1a* as a negative regulator of T_reg_ cell homeostasis is consistent with the conclusions of 2 previous reports [35,38]. However, *Hif1a* haploinsufficiency had little impact on the dysregulated IFNγ production in *miR-142*–deficient T_reg_ cells and failed to prevent development of fatal autoimmunity in *Foxp3*^*Cre*^*miR-142*^*fl/fl*^ mice. This outcome is not surprising given the fact that the T_reg_ cell number in *Foxp3*^*Cre*^*miR-142*^*fl/fl*^*Hif1a*^*+/fl*^ mice is only partially restored. The failure of *Hif1a* haploinsufficiency to fully rescue the T_reg_ cell defect in *Foxp3*^*Cre*^*miR-142*^*fl/fl*^ mice is probably linked to the abundance of miR-142-3p target genes in the IFNγ signaling pathway. The existence of multiple miR-142-3p targets likely makes the dysregulated state of IFNγ signaling in *miR-142*–deficient T_reg_ cells refractory to changes in the expression of a single target. This conclusion is supported by studies in miR-142-3p-deficient zebra danio, which display impaired myelopoiesis due to an aberrant activation of the IFNγ signaling pathway [17]. This developmental defect in miR-142-3p-deficient zebra danio could be rescued by a compound knockdown of *stat1a* and *irf1b* genes, whereas silencing of either factor alone was insufficient to restore normal neutrophil differentiation. Of note, based on the observations of dysregulated IFNγ signaling in *miR-142*–deficient immune cells from zebra danio and rodents, the role of *miR-142* in suppressing IFNγ signaling appears to be evolutionary conserved. In contrast, the function of *miR-142-Pde3b* signaling axis is unlikely to be evolutionary preserved, because miR-142-3p and miR-142-5p binding sites in the mouse Pde3b 3′ UTR are poorly conserved.

In summary, our results have established *miR-142* as a central regulator of T_reg_ cell development, homeostasis, and suppressive activity that mediates its function in T_reg_ cells in part by limiting IFNγ production and responsiveness. Besides advancing our understanding of the T_reg_ cell biology, these novel insights may open a new avenue for targeted pharmacological manipulation of T_reg_ cell activity in cancer immunotherapy and autoimmune disease settings.

## Materials and methods

### Mice

C57BL/6J (stock#000664), B6/CD45.1 (stock#002014), *Hif1a*^fl/fl^ (stock#007561), and *Foxp3*^YFP-Cre^ (stock#016959) mice were purchased from the Jackson Laboratory (Bar Harbor, Maine, USA). BALB/c mice were obtained from Charles River Laboratories (Wilmington, Massachusetts, USA). *miR-142*^fl/fl^ mice were described previously [14]. *Foxp3*^*Cre*^*miR-142*^*fl/fl*^ mice were generated by crossing *miR-142*^fl/fl^ mice with *Foxp3*^YFP-Cre^ deleter/reporter mice [21]. Mice were kept in a specific pathogen-free facility at the City of Hope Animal Resource Center, and all animal procedures were approved by the Institutional Animal Care and Use Committee (IACUC) of the City of Hope. Our approved animal protocols that are relevant for this work are the following: IACUC#13021, IACUC#13020, and IACUC#03008.

### Flow cytometry

For surface marker analysis, single cell suspensions from thymus, spleen, and peripheral lymph nodes (axillary, brachial, inguinal, and cervical) were treated with red blood cell (RBC) lysis buffer (BioLegend, San Diego, California, USA) to eliminate mature erythrocytes and then blocked with anti-CD16/CD32 antibody (Ab) to prevent nonspecific binding. Cells were stained with monoclonal fluorophore-conjugated antibodies against specific cell surface markers, such as anti-CD4 (clone RM4-5), anti-CD8α (clone 53–6.7), anti-CD25 (clone PC61), anti-CD44 (clone IM7), anti-CD62L (clone MEL-14), anti-CD45.1 (clone A20), anti-ICOS (clone 7E.17G9), anti-GITR (clone DTA-1), anti-CTLA-4 (clone UC10-4B9), anti-PD-1 (clone 29F.1A12), and anti-CD103 (clone 2E7) antibodies (all from BioLegend). Intracellular staining with anti-Foxp3 (clone FJK-16s; eBioscience, Santa Clara, California, USA) and anti-Hif1α (clone 241812; R&D Systems, Minneapolis, Minnesota, USA) antibodies was performed using fixed and permeabilized cells following the manufacturer’s protocol. For detection of phosphorylated Stat1 (pStat1), purified CD4^+^ T cells were first stimulated with IFNγ (100 ng/mL) for 24 hours, fixed with Cytofix buffer and permeabilized with Phosflow Perm Buffer III, and subsequently stained with anti-pStat1 (Y701; clone 4a; BD Biosciences, San Jose, California, USA) antibodies. For intracellular cytokine staining, T cells derived from spleen and lymph nodes or purified T_reg_ cells were first stimulated with phorbol 12-myristate 13-acetate (PMA; 50 ng/mL) and ionomycin (500 ng/mL) for 4 hours in the presence of monensin (2 μM) and then fixed, permeabilized, and stained following the manufacturer’s protocol (BioLegend). Data were acquired on Accuri C6 flow cytometer (BD Biosciences) and analyzed with FlowJo software BD Biosciences (San Jose, California, USA). Cell sorting for the analysis of *miR-142* expression in T-cell lineage was performed using FACSAriaIII (BD Biosciences) instrument.

All flow cytometry raw data generated in this study can be found at the Figshare data repository (https://figshare.com/projects/microRNA-142_guards_against_autoimmunity_by_controlling_Treg_cell_homeostasis_and_function/128960).

### Global transcriptome profiling by RNA-seq

CD4^+^YFP^+^ T_reg_ cells were sorted using BD FACSAria II machine from single-cell splenocyte suspensions derived from 8- to 11-week-old *Foxp3*^*Cre*^ and *Foxp3*^*Cre*^*miR-142*^*fl/fl*^ female mice (*n* = 3 per group). Total RNA from purified WT and *miR-142*–deficient T_reg_ cells was isolated using miRNeasy kit (QIAGEN, Hilden, Germany) and subjected to RNA-seq. RNA quality was determined with an Agilent Bioanalyzer (RNA integrity number (RIN) > 7.5 for all samples). Library was prepared according to the manufacturer’s protocol using Illumina TruSeq RNA Library Prep Kit v2 (San Diego, Califonia, USA) and subsequently loaded on an Illumina HiSeq 2500 for parallel sequencing. The 51 base pair single-ended sequence reads were mapped to the mouse reference genome (mm10) using the alignment program HISAT (https://daehwankimlab.github.io/hisat2). Gene expression was measured from alignment bam files by read counting function featureCounts in the Bioconductor package Rsubread (http://bioconductor.org/packages/Rsubread). The unstranded raw counts were then normalized using a trimmed mean of M values (TMM) method implemented in the Bioconductor package edgeR (https://bioconductor.org/packages/edgeR). A total of 11,658 genes having counts per million (CPM) values higher than 1 in at least 3 samples were included in the downstream differential expression analysis. Differentially expressed genes were tested using 3 statistical methods in edgeR, including the generalized linear model (GLM) quasi-likelihood F (QLF) test, the likelihood ratio (LR) test, and the exact test based on quantile-adjusted conditional maximum likelihood (qCML) methods. A complete list of genes and the statistical test results (*miR-142*–deficient (KO) versus *miR-142*–sufficient (WT) T_reg_ cells) are shown in S1 Table. Statistical *P* values were adjusted by Benjamini–Hochberg method for false discovery rate (FDR) controls. The volcano plot visualizing the distribution of differentially expressed genes was based on QLF test results. The RNA-seq raw data were deposited in the Gene Expression Omnibus under the accession number GSE190192.

### Pathway and GSEA

Differentially expressed genes passing the criterion of FDR lower than 0.05 for all 3 statistical methods mentioned above were subjected for pathway and GSEA using Enrichr (http://amp.pharm.mssm.edu/Enrichr) software algorithm [31]. Top ranked pathways across major databases, including Panther (http://www.pantherdb.org), Reactome (https://reactome.org), KEGG (https://www.genome.jp/kegg), and WikiPathways (https://www.wikipathways.org) were identified and are listed in S2 Table. In addition, GSEA analysis (http://software.broadinstitute.org/gsea/index.jsp)) [32] was performed with the CPM values from the 11,658 expressed genes rank-ordered by Signal2Noise metric. Gene ontology gene sets from Molecular Signatures Database (MSigDB) v7.1 were evaluated for the enrichment.

### Sylamer analysis

Analysis of miRNA seed enrichment in the 3′ UTRs of genes that are differentially expressed in *miR-142* KO T_reg_ cells was performed by the Web-based SylArray software algorithm (http://www.ebi.ac.uk/enright-srv/sylarray)) [26].

### In vitro T_reg_ cell suppression assay

CD4^+^ T cells were isolated from *Foxp3*^*Cre*^, *Foxp3*^*Cre*^*miR-142*^*fl/fl*^, and *Foxp3*^*Cre*^*miR-142*^*fl/fl*^*Hif1a*^*fl/fl*^ spleens using anti-CD4-biotin (clone GK1.5), anti-Biotin MicroBeads (Miltenyi Bergisch, Gladbach, North Rhine-Westphalia, Germany), LS columns (Miltenyi), and a MiniMACS Separator (Miltenyi). Enriched CD4^+^ T cells were sorted for YFP^+^(T_reg_) and YFP^−^(T_con_) cells using BD FACS Fusion. Moreover, 10^5^ *Foxp3*^*Cre*^ CD4^+^YFP^−^ T_con_ cells were labeled with 4μM CellTrace Violet (CTV) (Thermo Fisher, Waltham, Massachusetts, USA) at 37°C for 7.5 minutes and cultured with either *Foxp3*^*Cre*^*miR-142*^*fl/fl*^ or *Foxp3*^*Cre*^*miR-142*^*fl/fl*^*Hif1a*^*fl/fl*^ CD4^+^YFP^+^ T_reg_ cells at ratios of 1:0.5 and 1:0.125 in the presence of CD3ɛ/CD28-conjugated MACSiBead Particles (mouse T Cell Activation/Expansion Kit, Miltenyi) at a bead-to-cell ratio of 2:1. After 3 days of culturing, proliferation of *Foxp3*^*Cre*^ CD4^+^YFP^−^ T_con_ cells was measured by flow cytometry as CTV dilution.

### In vitro T_reg_ survival assay

CD4^+^ T cells isolated from *Foxp3*^*Cre*^ and *Foxp3*^*Cre*^*miR-142*^*fl/fl*^ spleens by EasySep Mouse CD4^+^ T Cell Isolation Kit (STEMCELL Technologies, Vancouver, British Columbia, Canada) were sorted for YFP expressing cells using BD FACSAria II instrument. CD4^+^YFP^+^ T_reg_ cells were stimulated with anti-CD3 (1 μg/mL) specific antibodies for 48 hours, stained with Annexin V-PE (BioLegend) and analyzed by flow cytometry. For the IFNγ-induced apoptosis of T_reg_ cells assay, splenocytes were cultured for 24 hours in the presence or absence of IFNγ (10 ng/mL) and then harvested and stained with Annexin V-PE for fluorescence activated cell sorting (FACS) analysis.

### In vivo labeling of T_reg_ cells with BrdU

*Foxp3*^*Cre*^ and *Foxp3*^*Cre*^*miR-142*^*fl/fl*^ mice were injected intraperitoneally with 1 mg of BrdU and splenic BrdU^+^ T_reg_ cells (CD4^+^YFP^+^) were quantified by flow cytometry 16 hours postinjection. Intracellular staining with anti-BrdU specific antibodies was performed using BrdU Flow Kit (BD Biosciences).

### aGVHD mouse model

BALB/c recipient mice were lethally irradiated (850 cGy) 8 to 10 hours prior to bone marrow transplantation (BMT) and subsequently transplanted via intravenous injection with T-cell–depleted bone marrow from C57BL/6J mice (TCD-BM, 2.5 × 10^6^ cells), Thy1.2^+^CD4^+^ T cells from C57BL/6J spleens (0.5 to 0.6 × 10^6^ cells), and CD4^+^YFP^+^ T_reg_ cells derived from either *Foxp3*^*Cre*^ or *Foxp3*^*Cre*^*miR-142*^*fl/fl*^ spleens (0.1 to 0.12 × 10^6^ cells). Body weight and severity of diarrhea in host mice were monitored and recorded for 15 days after BMT. Donor T_reg_ cell frequency in the host spleen was assessed by FACS on day 17 after transplantation.

### miRNA qRT-PCR

Total RNA was isolated using miRNeasy kit (QIAGEN) and reverse transcribed using the TaqMan MicroRNA Reverse Transcription Kit (Life Technologies, Carlsbad, California, USA). Mature miR-142-3p expression was assessed by TaqMan Real-Time miRNA assay (Life Technologies) and normalized to snoRNA234 levels.

### 3′ UTR luciferase reporter assays

DNA fragments encompassing WT and miR-142-3p seed mutated 3′ UTRs of mouse *Hif1a*, *Ifngr2*, and *Pde3b* genes were synthesized and cloned into pMIR-Report vector (Ambion, Austin, Texas, USA). The sequence complementary to the miR-142-3p.2 seed sequence (8-mer) in the *Ifngr2* 3′ UTR was mutated from 5′-AACACTAA-3′ to 5′-ACGTACCA-3′. The sequence complementary to the miR-142-3p.2 seed sequence (8-mer) in the *Hif1a* 3′ UTR was mutated from 5′- AACACTAA-3′ to 5′- ACGTACCA-3′. The sequence complementary to the miR-142-3p.1 seed sequence (6-mer) in the *Pde3b* 3′ UTR was mutated from 5′- CACTAC-3′ to 5′- CCAGCC-3′. To perform the 3′ UTR reporter assays, 10^5^ 293T cells in 24-well plates were transiently transfected using calcium phosphate with 10 ng of pMIR-Report-3′ UTR firefly luciferase plasmid, 20 ng of Renilla luciferase reporter plasmid (pRL-SV40; Promega, Madison, Wisconsin, USA), and 450 ng of either pMDH1-miR-142 or control pMDH1 vector (Addgene, Watertown, Massachusetts, USA). Cells were lysed 48 hours posttransfection, and the luciferase activities were assessed using Dual Luciferase Reporter assay (Promega).

### IFNγ ELISA

CD4^+^ T cells purified from *Foxp3*^*Cre*^ and *Foxp3*^*Cre*^*miR-142*^*fl/fl*^ splenocytes with the help of EasySep Mouse CD4^+^ T Cell Isolation Kit (STEMCELL Technologies) were sorted for YFP expressing cells using BD FACSAria II instrument. CD4^+^YFP^+^ T_reg_ cells (10^6^/mL) were stimulated with anti-CD3 (5 μg/mL) and anti-CD28 (2 μg/mL) specific antibodies for 48 hours in the presence of IL-2 (50 ng/mL) and cell culture supernatants were assessed for IFNγ production by sandwich ELISA. Briefly, 96-well flat-bottom Maxisorp (Thermo Fisher) plates were coated with anti-mouse IFNγ-specific capture antibodies (eBioscience, clone XMG1.2), and the captured mouse IFNγ was detected with biotin labeled anti-mouse IFNγ-specific antibodies (eBioscience, clone R4-6A2). Mouse recombinant IFNγ (eBioscience) was used as a standard to quantify the results.

### Histopathology

For histological sectioning, mouse tissues were collected and placed into 10% formalin, fixed for 24 hours, washed, and transferred to 70% ethanol before standard paraffin embedding. Tissue sections were stained with hematoxylin and eosin and examined by an experienced veterinary pathologist.

### Statistical analysis

All statistical analyses were performed using Prism 6 (GraphPad, San Diego, California, USA) software. Statistical analyses were performed using 2-tailed Student *t* test or ANOVA. Results were considered significant when *P* ≤ 0.05.

## Supporting information

S1 Fig *miR-142* expression in T-cell lineage and analysis of germline (*miR-142^−/−^*) and T_reg_ cell–specific (*Foxp3^Cre^miR-142^fl/fl^*) *miR-142* KO mice. (**A**) Impaired T_reg_ cell development in *miR-142*^−/−^ mice. Left panel, FACS analysis of WT and *miR-142*^−/−^ thymocytes with anti-CD4 and anti-Foxp3 antibodies. Foxp3^+^CD4^+^ T_reg_ cells are gated and numbers indicate the percentage of cells in the gate. Right panel, frequency of CD4^+^Foxp3^+^ T_reg_ cells in WT and *miR-142*^−/−^ thymi (*n* = 3 per group). (**B, C**) T_reg_ cell defect in the periphery of *miR-142*^−/−^ mice. FACS analysis of T_reg_ cells in spleen (**B**) and MLNs (**C**) from WT and *miR-142*^−/−^ mice. Right panel, frequency of CD4^+^Foxp3^+^ T_reg_ cells in WT and *miR-142*^−/−^ spleens and MLNs (*n* = 4 per group). (**D**) qRT-PCR analysis of mature miR-142-3p and miR-142-5p expression in different T-cell subsets purified from *Foxp3*^*Cre*^ mice (*n* = 2). DN, double negative CD4^-^CD8^-^ thymocytes; DP, double positive CD4^+^CD8^+^ thymocytes; SP, single positive CD4^+^YFP^−^ thymocytes; T_reg_, CD4^+^YFP^+^ T_reg_ cells from thymus and spleen, respectively; T_Naive_, naive CD4^+^YFP^−^CD62L^+^CD44^−^ splenic T cells; T_Activated_, activated CD4^+^YFP^−^CD62L^−^CD44^+^ splenic T cells. Expression level of miR-142-3p in DN population was arbitrarily set to 1. snoRNA234 levels were used for normalization. (**E**) qRT-PCR analysis of mature miR-142-3p expression in CD4^+^YFP^+^ T_reg_ and CD4^+^YFP^−^ T_eff_ cells purified from *Foxp3*^*Cre*^ and *Foxp3*^*Cre*^*miR-142*^*fl/fl*^ spleens. Expression level of miR-142-3p in CD4^+^YFP^+^ T_reg_ cells isolated from *Foxp3*^*Cre*^*miR-142*^*fl/fl*^ spleen was arbitrarily set to 1. snoRNA234 levels were used for normalization. Spleen (**F**) and thymus (**G**) weights in 8- to 11-week-old male *Foxp3*^*Cre*^ and *Foxp3*^*Cre*^*miR-142*^*fl/fl*^ mice (*n* ≥ 7 per group). Absolute cell counts in spleen (**H**), thymus (**I**), and peripheral lymph nodes (**J**) from *Foxp3*^*Cre*^ and *Foxp3*^*Cre*^*miR-142*^*fl/fl*^ mice (*n* = 6 per group). Results are shown as mean ± SD. *P* values were calculated using 2-tailed Student *t* test. **, *P* < 0.01; ***, *P* < 0.001; ****, *P* < 0.0001; NS, not significant. The underlying numerical raw data can be found in S1 Data file. The underlying flow cytometry raw data can be found at the Figshare repository. FACS, fluorescence activated cell sorting; KO, knockout; MLN, mesenteric lymph node; PLN, peripheral lymph node; SD, standard deviation; SP, spleen; T_reg_, regulatory T; WT, wild-type; YFP, yellow fluorescent protein.
(PDF)

S2 Fig Characterization of T_reg_ and T_eff_ cell defects in *Foxp3^Cre^miR-142^fl/fl^*mice. (**A**) FACS analysis of lymphocytes from PLNs (left panel) and thymus (right panel) of 12-week-old *Foxp3*^*Cre*^and *Foxp3*^*Cre*^*miR-142*^*fl/fl*^ mice with anti-CD4 and anti-Foxp3 specific antibodies. Foxp3^+^CD4^+^ T_reg_ cells are gated and numbers indicate the percentage of cells in the gate. (**B**) Frequency (left panel) and total number (right panel) of T_reg_ cells in PLNs and thymi of 12-week-old *Foxp3*^*Cre*^ (red bars) and *Foxp3*^*Cre*^*miR-142*^*fl/fl*^ (blue bars) mice (*n* = 3 per group). (**C**) FACS analysis of CD44 and CD62L expression on CD4^+^ (upper panel) and CD8^+^ (bottom panel) T cells from *Foxp3*^*Cre*^ and *Foxp3*^*Cre*^*miR-142*^*fl/fl*^ PLNs. Numbers indicate percentage of cells in the quadrants. (**D**) Frequency of CD44^−^CD62L^+^ (naive) and CD44^+^CD62L^−^ (activated) CD4^+^ (left panel) and CD8^+^ (right panel) T cells in *Foxp3*^*Cre*^ and *Foxp3*^*Cre*^*miR-142*^*fl/fl*^ PLNs (*n* = 6 per group). (**E**) Intracellular FACS analysis of IFNγ production by CD4^+^ (upper panel) and CD8^+^ (bottom panel) T cells from *Foxp3*^*Cre*^ and *Foxp3*^*Cre*^*miR-142*^*fl/fl*^ PLNs. IFNγ^+^ T cells are gated and numbers indicate percentage of cells in the gate. (**F**) Frequency of IFNγ-expressing CD4^+^ and CD8^+^ T cells in *Foxp3*^*Cre*^ (filled bars) and *Foxp3*^*Cre*^*miR-142*^*fl/fl*^ (open bars) PLNs (*n* = 4 per group). (**G**) Total CD4^+^ and CD8^+^ T cell counts in *Foxp3*^*Cre*^ (filled bars) and *Foxp3*^*Cre*^*miR-142*^*fl/fl*^ (open bars) PLNs (*n* = 6 per group). (**H**) Frequencies of IFNγ-, IL-4-, and IL-17-expressing CD4^+^ T cells isolated from *Foxp3*^*Cre*^ (filled bars) and *Foxp3*^*Cre*^*miR-142*^*fl/fl*^ (open bars) spleens (*n* ≥ 3 per group). Results are shown as mean ± SD. *P* values were calculated using 2-tailed Student *t* test. *, *P* < 0.05; **, *P* < 0.01; ***, *P* < 0.001; ****, *P* < 0.0001; NS, not significant. The underlying numerical raw data can be found in S1 Data file. The underlying flow cytometry raw data can be found at the Figshare repository. FACS, fluorescence activated cell sorting; IFNγ, interferon gamma; IL, interleukin; PLN, peripheral lymph node; SD, standard deviation; T_reg_, regulatory T.
(PDF)

S3 Fig Immunophenotyping of *miR-142*–deficient T_reg_ cells. (**A**) FACS analysis of T_reg_ suppression activity in vitro. Purified CD4^+^ T_conv_ cells were loaded with CTV dye and incubated with FACS-sorted T_reg_ cells from *Foxp3*^*Cre*^ (red line) and *Foxp3*^*Cre*^*miR-142*^*fl/fl*^ (blue line) spleens in the presence of beads coated with anti-CD3 and anti-CD28 specific antibodies. Several T_reg_ to T_conv_ cell ratios were shown as indicated. (**B**) Left panel, FACS analysis of Annexin V-stained CD4^+^YFP^+^ T_reg_ cells from *Foxp3*^*Cre*^ (WT) or *Foxp3*^*Cre*^*miR-142*^*fl/fl*^ (KO) spleens. Right panel, MFI of Annexin V staining on T_reg_ cells from *Foxp3*^*Cre*^ (WT) or *Foxp3*^*Cre*^*miR-142*^*fl/fl*^ (KO) spleens. (**C**) Frequency of splenic YFP^+^ T_reg_ cells in female *Foxp3*^Cre/WT^*miR-142*^*+/+*^ and *Foxp3*^Cre/WT^*miR-142*^*fl/fl*^ mice. (**D**) MFI of GITR, CD25, CD103, PD-1, CTLA-4, and ICOS expression on splenic CD4^+^YFP^+^ T_reg_ cells from *Foxp3*^*Cre*^ (WT) or *Foxp3*^*Cre*^*miR-142*^*fl/fl*^ (KO) mice. Results are shown as mean ± SD. *P* values were calculated using 2-tailed Student *t* test *, *P* < 0.05; **, *P* < 0.01; NS, not significant. The underlying numerical raw data can be found in S1 Data file. The underlying flow cytometry raw data can be found at the Figshare repository. CTV, cell trace violet; FACS, fluorescence activated cell sorting; KO, knockout; MFI, mean fluorescence intensity; T_reg_, regulatory T; WT, wild-type; YFP, yellow fluorescent protein.
(PDF)

S4 Fig Gene expression signatures in *miR-142*–deficient T_reg_ cells. Heatmap visualization of differentially expressed cytokine, chemokine, and immune receptor genes (**A**) and T_reg_ cell signature genes (**B**). Genes highlighted in red are putative miR-142-3p targets. (**C**) Standard enrichment plot demonstrating the enrichment of up-regulated genes in *miR-142*–deficient T_reg_ cells from gene ontology term “regulation of response to interferon gamma” using the GSEA tool. (**D**) ELISA analysis of IFNγ production by *Foxp3*^*Cre*^ (WT) and *Foxp3*^*Cre*^*miR-142*^*fl/fl*^ (KO) T_reg_ cells (*n* = 4 per group). Purified CD4^+^YFP^+^ T_reg_ cells (10^6^/mL) were stimulated with anti-CD3 (5 μg/ml) and anti-CD28 (2 μg/ml) antibodies in the presence of IL-2 (50 ng/ml) for 48 hours. (**E**) MFI of phospho-Stat1(Y701) levels in *Foxp3*^*Cre*^ (WT; *n* = 6) and *Foxp3*^*Cre*^*miR-142*^*fl/fl*^ (KO; *n* = 5) T_reg_ cells. *P* values were calculated using 2-tailed Student *t* test. *, *P* < 0.05; **, *P* < 0.01. The underlying raw data can be found in S1 Data file. GSEA, Gene Set Enrichment Analysis; IFNγ, interferon gamma; IL, interleukin; KO, knockout; MFI, mean fluorescence intensity; T_reg_, regulatory T; WT, wild-type; YFP, yellow fluorescent protein.
(PDF)

S5 Fig Characterization of the molecular mechanism by which *miR-142* controls T_reg_ cell homeostasis and function. (**A**) Diagrams (top left) and sequence alignments (top right) of putative miR-142-3p binding sites in the 3′ UTRs of *Stat1*, *Ifngr2*, *Irf1*, *Gbp3*, and *Pde3b* genes. HITS-CLIP analysis of Ago2 binding to the 3′ UTRs of *Ifngr2* (bottom-left) and *Gbp3* (bottom-right) genes in WT (blue plot) and *miR-155* KO (yellow plot) activated CD4^+^ T cells. Sequences corresponding to miR-142-3p binding sites are labeled by arrows. (**B**) Validation of *Pde3b* and *Ifngr2* as direct miR-142-3p targets by the 3′ UTR luciferase reporter assay (*n* = 2). Relative expression of WT and miR-142-3p seed mutated *Pde3b* and *Ifngr2* 3′ UTR reporter constructs upon cotransfection with either miR-142 precursor expressing plasmid or empty vector control. Expression of WT *Pde3b* and *Ifngr2* 3′ UTR reporters in the presence of empty vector were set to 1. (**C**) MFI of Hif1α in *Foxp3*^*Cre*^ (WT; red bars) and *Foxp3*^*Cre*^*miR-142*^*fl/fl*^ (KO; blue bars) CD4^+^Foxp3^+^ T_reg_ and CD4^+^Foxp3^-^ T_conv_ cells (*n* = 6 per group). (**D**) Left panel, FACS analysis of IFNγR2 expression in CD4^+^YFP^+^ T cells from *Foxp3*^*Cre*^ (red line) and *Foxp3*^*Cre*^*miR-142*^*fl/fl*^ (blue line) spleens; right panel, MFI of IFNγR2 in *Foxp3*^*Cre*^ (WT; red bars) and *Foxp3*^*Cre*^*miR-142*^*fl/fl*^ (KO; blue bars) CD4^+^YFP^+^ T_reg_ cells. (**E**) Schematic diagram and sequence conservation of 2 miR-142-5p and one miR-142-3p binding sites in the 3′ UTR of mouse *Pde3b* gene as determined by the TargetScan algorithm. (**F**) Sequence alignment of miR-142-3p binding sites in mouse Pde3b-WT and Pde3b-MUT 3′ UTR reporter constructs. Results are shown as mean ± SD. *P* values were calculated using 2-tailed Student *t* test. *, *P* < 0.05; **, *P* < 0.01; ***, *P* < 0.001; NS, not significant. The underlying numerical raw data can be found in S1 Data file. The underlying flow cytometry raw data can be found at the Figshare repository. FACS, fluorescence activated cell sorting; IFNγ, interferon gamma; KO, knockout; MFI, mean fluorescence intensity; SD, standard deviation; T_reg_, regulatory T; WT, wild-type; YFP, yellow fluorescent protein.
(PDF)

S6 Fig Phenotypic analysis of *Foxp3^Cre^miR-142^fl/fl^Hif1a^+/fl^* and *Foxp3^Cre^miR-142^fl/fl^Hif1a^fl/fl^* mice. (**A**) Relative Hif1α protein expression in *Foxp3*^*Cre*^ (WT; red bar), *Foxp3*^*Cre*^*miR-142*^*fl/fl*^ (KO; blue bar), and *Foxp3*^*Cre*^*miR-142*^*fl/fl*^*Hif1a*^+/fl^ (HET; green bar) T_reg_ cells. The Hif1α levels in WT T_reg_ cells were arbitrarily set to 1. (**B**) Frequency of CD4^+^Foxp3^+^ T_reg_ cells in splenic lymphocytes from *Foxp3*^*Cre*^ (WT; red bar; *n* = 7), *Foxp3*^*Cre*^*miR-142*^*fl/fl*^ (KO; blue bar; *n* = 5) and *Foxp3*^*Cre*^*miR-142*^*fl/fl*^*Hif1a*^+/fl^ (HET; green bar; *n* = 3) mice. (**C**) Kaplan–Meier survival curves for *Foxp3*^*Cre*^ (red line; *n* = 27), *Foxp3*^*Cre*^*miR-142*^*fl/fl*^ (blue line; *n* = 27), and *Foxp3*^*Cre*^*miR-142*^*fl/fl*^*Hif1a*^+/fl^ (green line; *n* = 8) mice. Analysis of IFNγ production (**D**) and Stat1 activation (**E**) in T_reg_ cells from *Foxp3*^*Cre*^*miR-142*^*fl/fl*^*Hif1a*^+/fl^ mice. Left panels, intracellular FACS analysis of splenic CD4^+^Foxp3^+^ T_reg_ cells from *Foxp3*^*Cre*^ (red line; WT), *Foxp3*^*Cre*^*miR-142*^*fl/fl*^ (blue line; KO) and *Foxp3*^*Cre*^*miR-142*^*fl/fl*^*Hif1a*^+/fl^ (green line; HET) mice with anti-IFNγ (**D**) and anti-pStat1 (Y701) (**E**) antibodies. Right panels, MFI of IFNγ and phospho-Stat1 (pSTAT1) in *Foxp3*^*Cre*^ (WT; red bar), *Foxp3*^*Cre*^*miR-142*^*fl/fl*^ (KO; blue bar) and *Foxp3*^*Cre*^*miR-142*^*fl/fl*^*Hif1a*^+/fl^ (HET; green bar) T_reg_ cells. (**F, G**) FACS analysis of immunosuppressive activity of T_reg_ cells derived from *Foxp3*^*Cre*^ (WT; red dot), *Foxp3*^*Cre*^*miR-142*^*fl/fl*^ (KO; blue dot) and *Foxp3*^*Cre*^*miR-142*^*fl/fl*^*Hif1a*^*fl*/fl^ (DKO; green dot) mice (*n* = 3) in vitro. Several T_reg_ to T_conv_ cell ratios were analyzed as indicated in the graph. Unstimulated T_conv_ cells were used as control. Representative FACS plot analysis is shown in G. (**H**) FACS analysis of CD44 and CD62L expression in splenic CD4^+^ T cells from *Foxp3*^*Cre*^, *Foxp3*^*Cre*^*miR-142*^*fl/fl*^*Hif1a*^*+/fl*^, and *Foxp3*^*Cre*^*miR-142*^*fl/fl*^*Hif1a*^*fl/fl*^ mice. Numbers indicate percentage of cells in the quadrants. Results are shown as mean ± SD. *P* values were calculated using 2-tailed Student *t* test. *, *P* < 0.05; **, *P* < 0.01; ****, *P* < 0.0001; NS, not significant. The underlying numerical raw data can be found in S1 Data file. The underlying flow cytometry raw data can be found at the Figshare repository. DKO, double knockout; FACS, fluorescence activated cell sorting; IFNγ, interferon gamma; KO, knockout; MFI, mean fluorescence intensity; pStat1, phosphorylated Stat1; SD, standard deviation; T_reg_, regulatory T; WT, wild-type.
(PDF)

S1 Data Data underlying figures. (XLSX)

S1 Table Differentially expressed genes in *miR-142*–deficient T_reg_ cells. T_reg_, regulatory T.
(XLSX)

S2 Table Pathway enrichment analysis by Enrichr. (XLSX)

## Abbreviations

Ab: antibody
aGVHD: acute graft-versus-host disease
BMT: bone marrow transplantation
BrdU: 5-bromo-2-deoxyuridine
CPM: counts per million
CTLA4: cytotoxic T-lymphocyte associated protein 4
CTV: CellTrace Violet
DC: dendritic cell
DP: double positive
FACS: fluorescence activated cell sorting
FDR: false discovery rate
GITR: glucocorticoid-induced tumor necrosis factor receptor family-related gene
GLM: generalized linear model
GSEA: Gene Set Enrichment Analysis
Hif1a: hypoxia-induced factor 1 alpha
HITS-CLIP: high-throughput sequencing of RNAs isolated by cross-linking immunoprecipitation
IACUC: Institutional Animal Care and Use Committee
ICOS: inducible T-cell co-stimulator
IFNγ: interferon gamma
IL: interleukin
KO: knockout
LR: likelihood ratio
miRNA: microRNA
PD-1: programmed death-1
PMA: phorbol 12-myristate 13-acetate
pStat1: phosphorylated Stat1
pT_reg_: peripheral Treg
qCML: quantile-adjusted conditional maximum likelihood
QLF: quasi-likelihood F
RBC: red blood cell
RIN: RNA integrity number
RNA-seq: RNA sequencing
SP: single positive
T_conv_: conventional T
TCR: T cell receptor
T_eff_: T effector
Th1: T helper cell 1
T_reg_: regulatory T
TMM: trimmed mean of M values
VHL: von Hippel–Lindau
WT: wild-type
YFP: yellow fluorescent protein
