# Supplementary figures and images for: microRNA-142 guards against autoimmunity by controlling Treg cell homeostasis and function
Source: PLoS Biol. 2022 Feb 18;20(2):e3001552. doi: 10.1371/journal.pbio.3001552 (PMC8893712; doi:10.1371/journal.pbio.3001552)

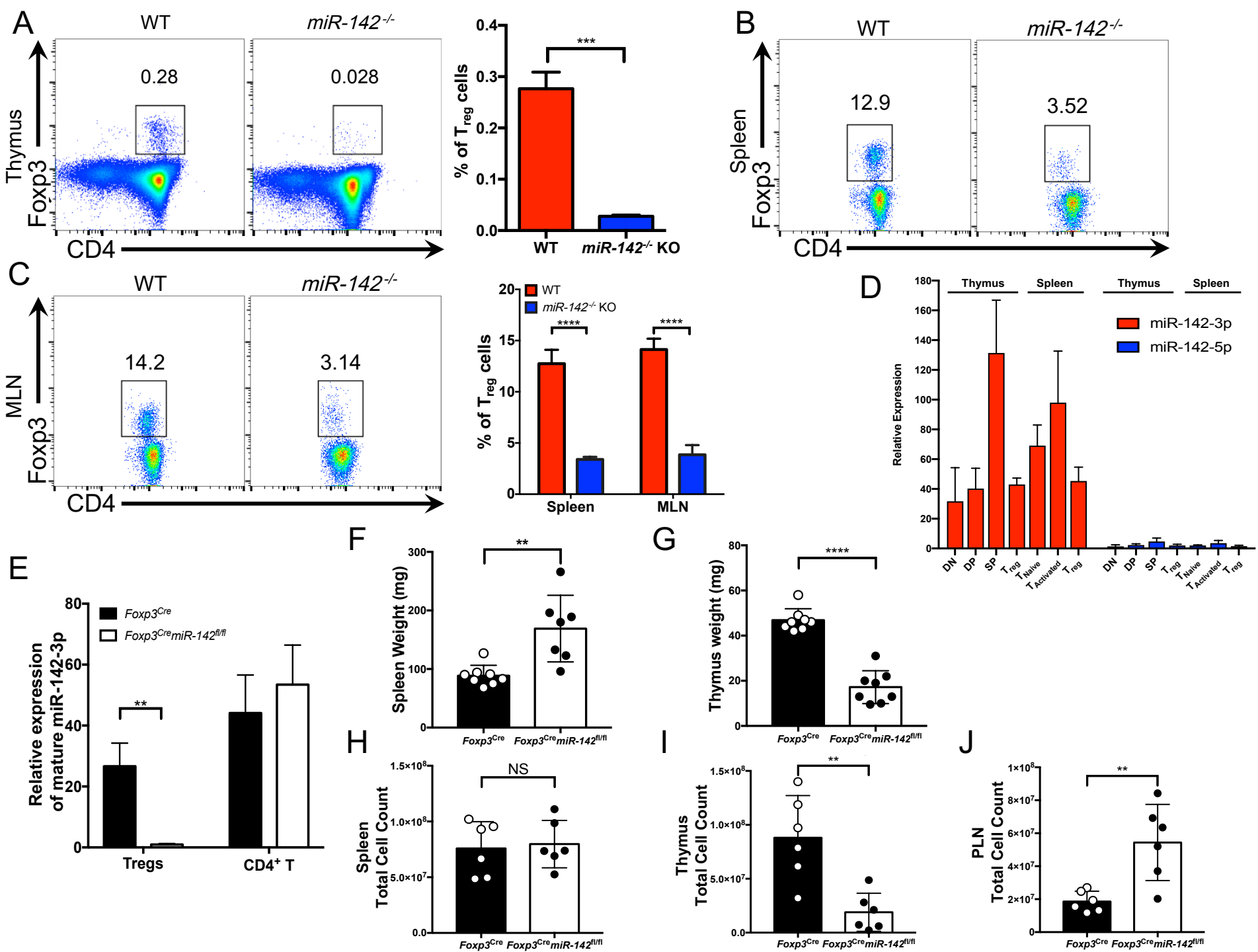

Supplement: S1 Fig — (A) Impaired Treg cell development in miR-142−/− mice. Left panel, FACS analysis of WT and miR-142−/− thymocytes with anti-CD4 and anti-Foxp3 antibodies. Foxp3+CD4+ Treg cells are gated and numbers indicate the percentage of cells in the gate. Right panel, frequency of CD4+Foxp3+ Treg cells in WT and miR-142−/− thymi (n = 3 per group). (B, C) Treg cell defect in the periphery of miR-142−/− mice. FACS analysis of Treg cells in spleen (B) and MLNs (C) from WT and miR-142−/− mice. Right panel, frequency of CD4+Foxp3+ Treg cells in WT and miR-142−/− spleens and MLNs (n = 4 per group). (D) qRT-PCR analysis of mature miR-142-3p and miR-142-5p expression in different T-cell subsets purified from Foxp3Cre mice (n = 2). DN, double negative CD4-CD8- thymocytes; DP, double positive CD4+CD8+ thymocytes; SP, single positive CD4+YFP− thymocytes; Treg, CD4+YFP+ Treg cells from thymus and spleen, respectively; TNaive, naive CD4+YFP−CD62L+CD44− splenic T cells; TActivated, activated CD4+YFP−CD62L−CD44+ splenic T cells. Expression level of miR-142-3p in DN population was arbitrarily set to 1. snoRNA234 levels were used for normalization. (E) qRT-PCR analysis of mature miR-142-3p expression in CD4+YFP+ Treg and CD4+YFP− Teff cells purified from Foxp3Cre and Foxp3CremiR-142fl/fl spleens. Expression level of miR-142-3p in CD4+YFP+ Treg cells isolated from Foxp3CremiR-142fl/fl spleen was arbitrarily set to 1. snoRNA234 levels were used for normalization. Spleen (F) and thymus (G) weights in 8- to 11-week-old male Foxp3Cre and Foxp3CremiR-142fl/fl mice (n ≥ 7 per group). Absolute cell counts in spleen (H), thymus (I), and peripheral lymph nodes (J) from Foxp3Cre and Foxp3CremiR-142fl/fl mice (n = 6 per group). Results are shown as mean ± SD. P values were calculated using 2-tailed Student t test. **, P < 0.01; ***, P < 0.001; ****, P < 0.0001; NS, not significant. The underlying numerical raw data can be found in S1 Data file. The underlying flow cytometry raw data can be found at the Fig [file pbio.3001552.s001.pdf]

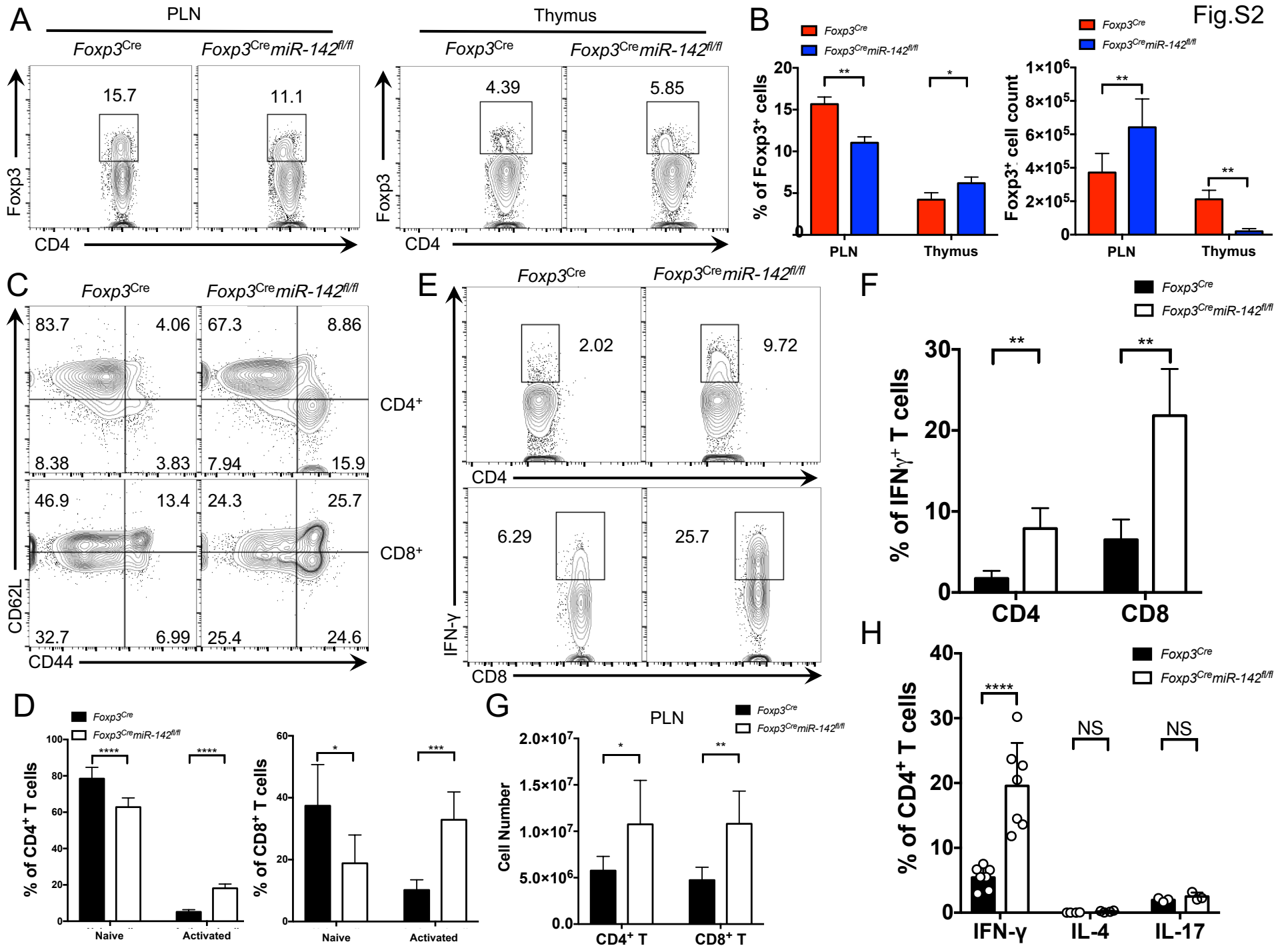

Supplement: S2 Fig — (A) FACS analysis of lymphocytes from PLNs (left panel) and thymus (right panel) of 12-week-old Foxp3Creand Foxp3CremiR-142fl/fl mice with anti-CD4 and anti-Foxp3 specific antibodies. Foxp3+CD4+ Treg cells are gated and numbers indicate the percentage of cells in the gate. (B) Frequency (left panel) and total number (right panel) of Treg cells in PLNs and thymi of 12-week-old Foxp3Cre (red bars) and Foxp3CremiR-142fl/fl (blue bars) mice (n = 3 per group). (C) FACS analysis of CD44 and CD62L expression on CD4+ (upper panel) and CD8+ (bottom panel) T cells from Foxp3Cre and Foxp3CremiR-142fl/fl PLNs. Numbers indicate percentage of cells in the quadrants. (D) Frequency of CD44−CD62L+ (naive) and CD44+CD62L− (activated) CD4+ (left panel) and CD8+ (right panel) T cells in Foxp3Cre and Foxp3CremiR-142fl/fl PLNs (n = 6 per group). (E) Intracellular FACS analysis of IFNγ production by CD4+ (upper panel) and CD8+ (bottom panel) T cells from Foxp3Cre and Foxp3CremiR-142fl/fl PLNs. IFNγ+ T cells are gated and numbers indicate percentage of cells in the gate. (F) Frequency of IFNγ-expressing CD4+ and CD8+ T cells in Foxp3Cre (filled bars) and Foxp3CremiR-142fl/fl (open bars) PLNs (n = 4 per group). (G) Total CD4+ and CD8+ T cell counts in Foxp3Cre (filled bars) and Foxp3CremiR-142fl/fl (open bars) PLNs (n = 6 per group). (H) Frequencies of IFNγ-, IL-4-, and IL-17-expressing CD4+ T cells isolated from Foxp3Cre (filled bars) and Foxp3CremiR-142fl/fl (open bars) spleens (n ≥ 3 per group). Results are shown as mean ± SD. P values were calculated using 2-tailed Student t test. *, P < 0.05; **, P < 0.01; ***, P < 0.001; ****, P < 0.0001; NS, not significant. The underlying numerical raw data can be found in S1 Data file. The underlying flow cytometry raw data can be found at the Figshare repository. FACS, fluorescence activated cell sorting; IFNγ, interferon gamma; IL, interleukin; PLN, peripheral lymph node; SD, standard deviation; Treg, regulatory T. (PDF) [file pbio.3001552.s002.pdf]

A

Cytokines, Chemokines and Immune receptors

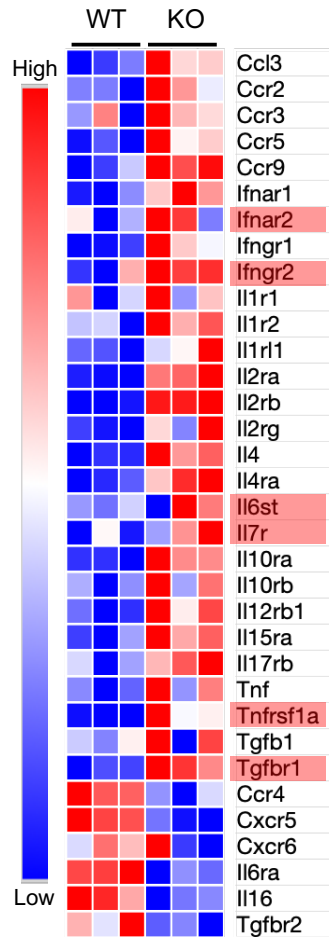

B

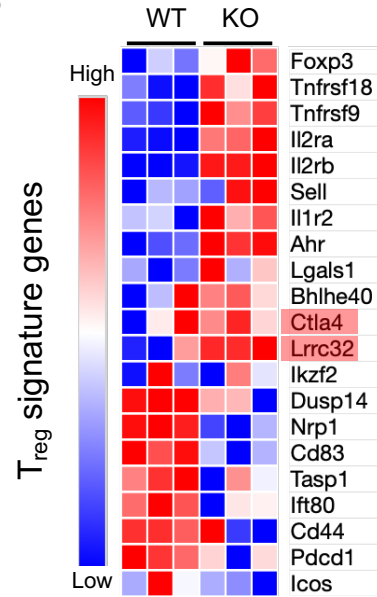

C

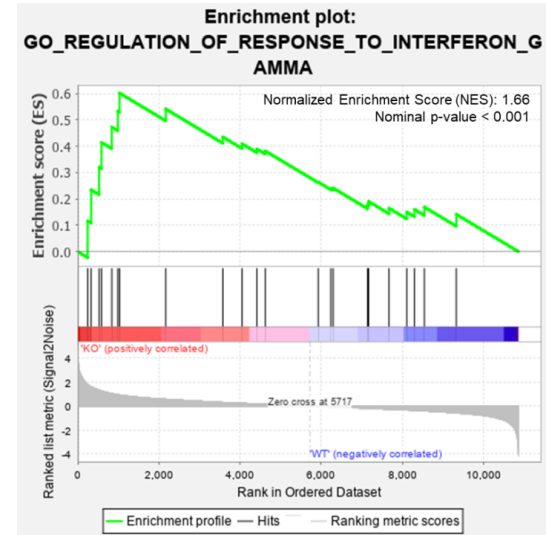

D

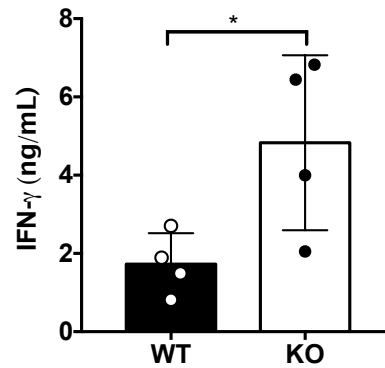

E

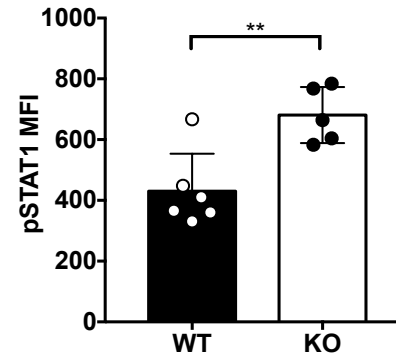

Supplement: S4 Fig — Heatmap visualization of differentially expressed cytokine, chemokine, and immune receptor genes (A) and Treg cell signature genes (B). Genes highlighted in red are putative miR-142-3p targets. (C) Standard enrichment plot demonstrating the enrichment of up-regulated genes in miR-142–deficient Treg cells from gene ontology term “regulation of response to interferon gamma” using the GSEA tool. (D) ELISA analysis of IFNγ production by Foxp3Cre (WT) and Foxp3CremiR-142fl/fl (KO) Treg cells (n = 4 per group). Purified CD4+YFP+ Treg cells (106/mL) were stimulated with anti-CD3 (5 μg/ml) and anti-CD28 (2 μg/ml) antibodies in the presence of IL-2 (50 ng/ml) for 48 hours. (E) MFI of phospho-Stat1(Y701) levels in Foxp3Cre (WT; n = 6) and Foxp3CremiR-142fl/fl (KO; n = 5) Treg cells. P values were calculated using 2-tailed Student t test. *, P < 0.05; **, P < 0.01. The underlying raw data can be found in S1 Data file. GSEA, Gene Set Enrichment Analysis; IFNγ, interferon gamma; IL, interleukin; KO, knockout; MFI, mean fluorescence intensity; Treg, regulatory T; WT, wild-type; YFP, yellow fluorescent protein. (PDF) [file pbio.3001552.s004.pdf]

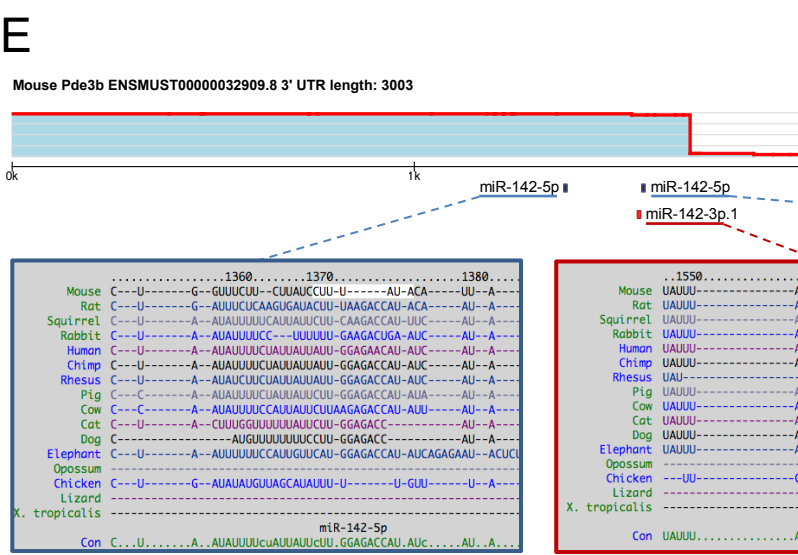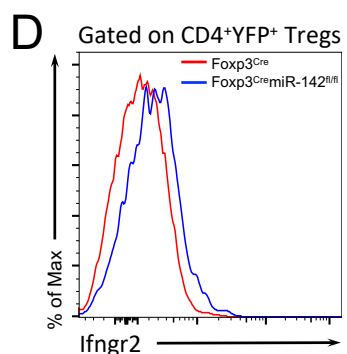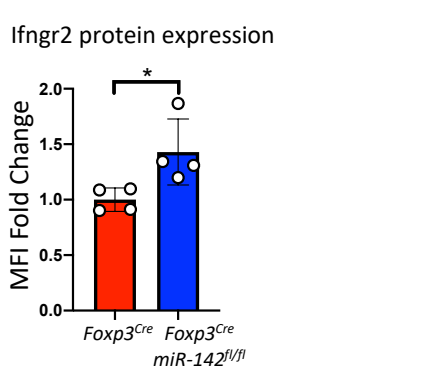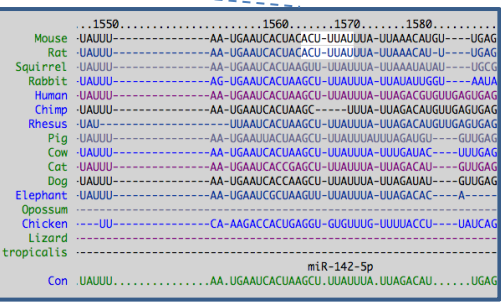

Supplement: S5 Fig — (A) Diagrams (top left) and sequence alignments (top right) of putative miR-142-3p binding sites in the 3′ UTRs of Stat1, Ifngr2, Irf1, Gbp3, and Pde3b genes. HITS-CLIP analysis of Ago2 binding to the 3′ UTRs of Ifngr2 (bottom-left) and Gbp3 (bottom-right) genes in WT (blue plot) and miR-155 KO (yellow plot) activated CD4+ T cells. Sequences corresponding to miR-142-3p binding sites are labeled by arrows. (B) Validation of Pde3b and Ifngr2 as direct miR-142-3p targets by the 3′ UTR luciferase reporter assay (n = 2). Relative expression of WT and miR-142-3p seed mutated Pde3b and Ifngr2 3′ UTR reporter constructs upon cotransfection with either miR-142 precursor expressing plasmid or empty vector control. Expression of WT Pde3b and Ifngr2 3′ UTR reporters in the presence of empty vector were set to 1. (C) MFI of Hif1α in Foxp3Cre (WT; red bars) and Foxp3CremiR-142fl/fl (KO; blue bars) CD4+Foxp3+ Treg and CD4+Foxp3- Tconv cells (n = 6 per group). (D) Left panel, FACS analysis of IFNγR2 expression in CD4+YFP+ T cells from Foxp3Cre (red line) and Foxp3CremiR-142fl/fl (blue line) spleens; right panel, MFI of IFNγR2 in Foxp3Cre (WT; red bars) and Foxp3CremiR-142fl/fl (KO; blue bars) CD4+YFP+ Treg cells. (E) Schematic diagram and sequence conservation of 2 miR-142-5p and one miR-142-3p binding sites in the 3′ UTR of mouse Pde3b gene as determined by the TargetScan algorithm. (F) Sequence alignment of miR-142-3p binding sites in mouse Pde3b-WT and Pde3b-MUT 3′ UTR reporter constructs. Results are shown as mean ± SD. P values were calculated using 2-tailed Student t test. *, P < 0.05; **, P < 0.01; ***, P < 0.001; NS, not significant. The underlying numerical raw data can be found in S1 Data file. The underlying flow cytometry raw data can be found at the Figshare repository. FACS, fluorescence activated cell sorting; IFNγ, interferon gamma; KO, knockout; MFI, mean fluorescence intensity; SD, standard deviation; Treg, regulatory T; WT, wild-type; YFP, yellow fluorescent prot [file pbio.3001552.s005.pdf]

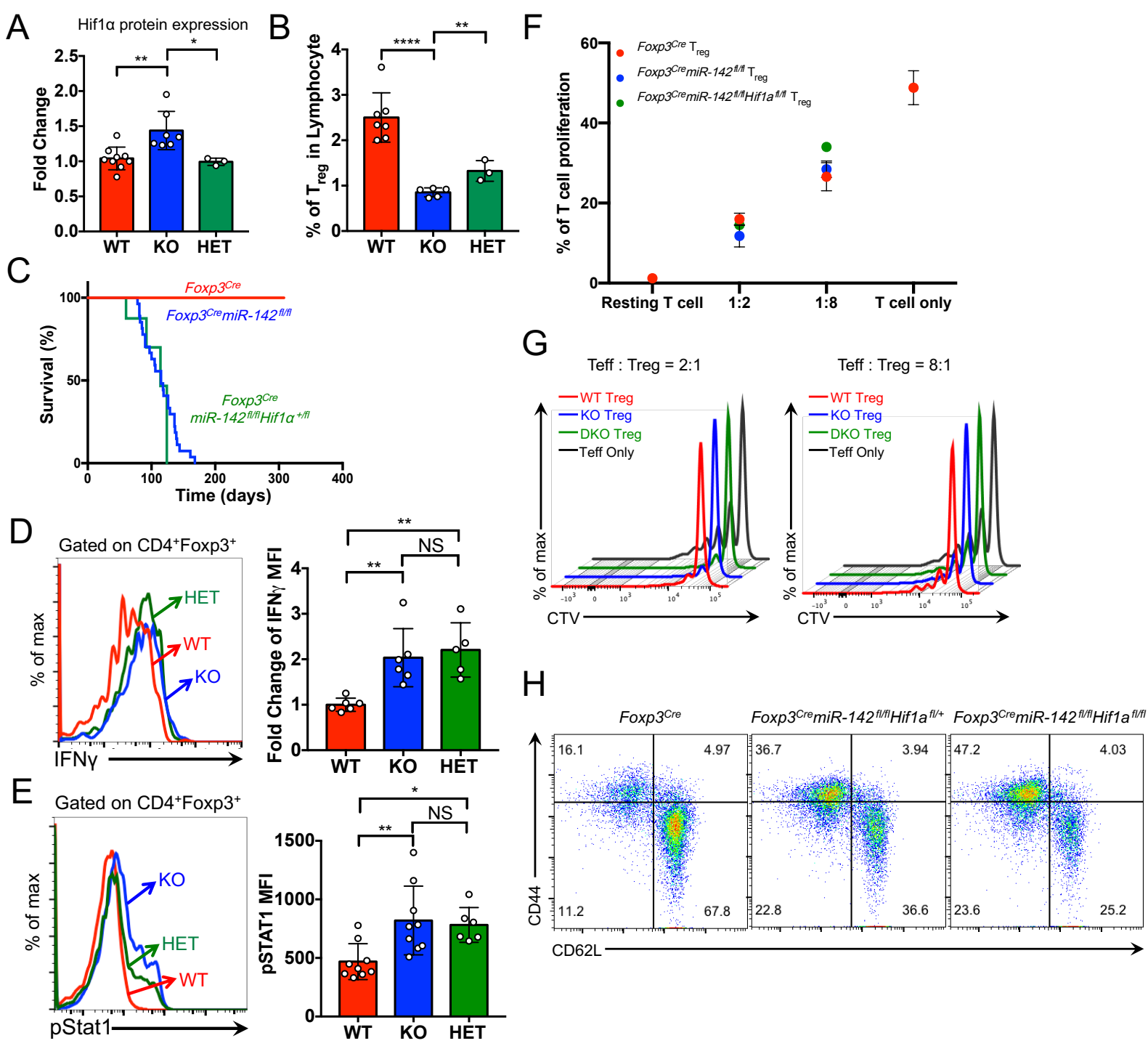

Supplement: S6 Fig — (A) Relative Hif1α protein expression in Foxp3Cre (WT; red bar), Foxp3CremiR-142fl/fl (KO; blue bar), and Foxp3CremiR-142fl/flHif1a+/fl (HET; green bar) Treg cells. The Hif1α levels in WT Treg cells were arbitrarily set to 1. (B) Frequency of CD4+Foxp3+ Treg cells in splenic lymphocytes from Foxp3Cre (WT; red bar; n = 7), Foxp3CremiR-142fl/fl (KO; blue bar; n = 5) and Foxp3CremiR-142fl/flHif1a+/fl (HET; green bar; n = 3) mice. (C) Kaplan–Meier survival curves for Foxp3Cre (red line; n = 27), Foxp3CremiR-142fl/fl (blue line; n = 27), and Foxp3CremiR-142fl/flHif1a+/fl (green line; n = 8) mice. Analysis of IFNγ production (D) and Stat1 activation (E) in Treg cells from Foxp3CremiR-142fl/flHif1a+/fl mice. Left panels, intracellular FACS analysis of splenic CD4+Foxp3+ Treg cells from Foxp3Cre (red line; WT), Foxp3CremiR-142fl/fl (blue line; KO) and Foxp3CremiR-142fl/flHif1a+/fl (green line; HET) mice with anti-IFNγ (D) and anti-pStat1 (Y701) (E) antibodies. Right panels, MFI of IFNγ and phospho-Stat1 (pSTAT1) in Foxp3Cre (WT; red bar), Foxp3CremiR-142fl/fl (KO; blue bar) and Foxp3CremiR-142fl/flHif1a+/fl (HET; green bar) Treg cells. (F, G) FACS analysis of immunosuppressive activity of Treg cells derived from Foxp3Cre (WT; red dot), Foxp3CremiR-142fl/fl (KO; blue dot) and Foxp3CremiR-142fl/flHif1afl/fl (DKO; green dot) mice (n = 3) in vitro. Several Treg to Tconv cell ratios were analyzed as indicated in the graph. Unstimulated Tconv cells were used as control. Representative FACS plot analysis is shown in G. (H) FACS analysis of CD44 and CD62L expression in splenic CD4+ T cells from Foxp3Cre, Foxp3CremiR-142fl/flHif1a+/fl, and Foxp3CremiR-142fl/flHif1afl/fl mice. Numbers indicate percentage of cells in the quadrants. Results are shown as mean ± SD. P values were calculated using 2-tailed Student t test. *, P < 0.05; **, P < 0.01; ****, P < 0.0001; NS, not significant. The underlying numerical raw data can be found in S1 Data file. The underlying flow cytometry raw data [file pbio.3001552.s006.pdf]
