# Supplementary material for: microRNA-142 guards against autoimmunity by controlling Treg cell homeostasis and function
Source: PLoS Biol. 2022 Feb 18;20(2):e3001552. doi: 10.1371/journal.pbio.3001552 (PMC8893712; doi:10.1371/journal.pbio.3001552)
Supplement: S3 Fig — (A) FACS analysis of Treg suppression activity in vitro. Purified CD4+ Tconv cells were loaded with CTV dye and incubated with FACS-sorted Treg cells from Foxp3Cre (red line) and Foxp3CremiR-142fl/fl (blue line) spleens in the presence of beads coated with anti-CD3 and anti-CD28 specific antibodies. Several Treg to Tconv cell ratios were shown as indicated. (B) Left panel, FACS analysis of Annexin V-stained CD4+YFP+ Treg cells from Foxp3Cre (WT) or Foxp3CremiR-142fl/fl (KO) spleens. Right panel, MFI of Annexin V staining on Treg cells from Foxp3Cre (WT) or Foxp3CremiR-142fl/fl (KO) spleens. (C) Frequency of splenic YFP+ Treg cells in female Foxp3Cre/WTmiR-142+/+ and Foxp3Cre/WTmiR-142fl/fl mice. (D) MFI of GITR, CD25, CD103, PD-1, CTLA-4, and ICOS expression on splenic CD4+YFP+ Treg cells from Foxp3Cre (WT) or Foxp3CremiR-142fl/fl (KO) mice. Results are shown as mean ± SD. P values were calculated using 2-tailed Student t test *, P < 0.05; **, P < 0.01; NS, not significant. The underlying numerical raw data can be found in S1 Data file. The underlying flow cytometry raw data can be found at the Figshare repository. CTV, cell trace violet; FACS, fluorescence activated cell sorting; KO, knockout; MFI, mean fluorescence intensity; Treg, regulatory T; WT, wild-type; YFP, yellow fluorescent protein. (PDF) [file pbio.3001552.s003.pdf]

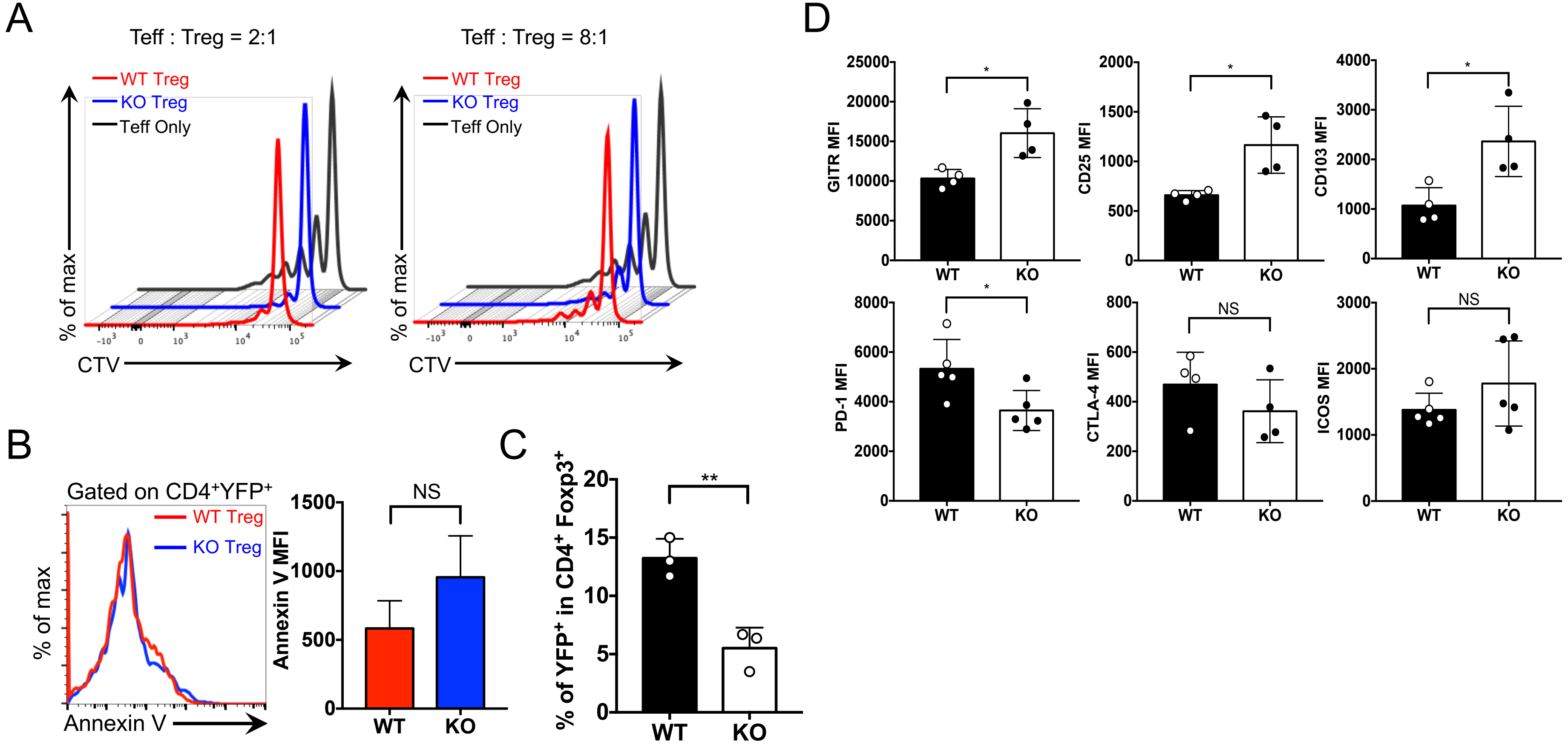
A
Teff : Treg = 2:1
Teff : Treg = 8:1
WT Treg
KO Treg
Teff Only
% of max
CTV
B
Gated on CD4+YFP+
WT Treg
KO Treg
% of max
Annexin V
Annexin V MFI
NS
WT
KO
C
% of YFP+ in CD4+ Foxp3+
**
WT
KO
D
GITR MFI
CD25 MFI
CD103 MFI
PD-1 MFI
CTLA-4 MFI
ICOS MFI
*
NS
WT
KO
